# Early Triassic Marine Biotic Recovery: The Predators' Perspective

**DOI:** 10.1371/journal.pone.0088987

**Published:** 2014-03-19

**Authors:** Torsten M. Scheyer, Carlo Romano, Jim Jenks, Hugo Bucher

**Affiliations:** 1 Paläontologisches Institut und Museum, Universität Zürich, Zürich, Switzerland; 2 West Jordan, Utah, United States of America; 3 New Mexico Museum of Natural History and Science, Albuquerque, New Mexico, United States of America; Raymond M. Alf Museum of Paleontology, United States of America

## Abstract

Examining the geological past of our planet allows us to study periods of severe climatic and biological crises and recoveries, biotic and abiotic ecosystem fluctuations, and faunal and floral turnovers through time. Furthermore, the recovery dynamics of large predators provide a key for evaluation of the pattern and tempo of ecosystem recovery because predators are interpreted to react most sensitively to environmental turbulences. The end-Permian mass extinction was the most severe crisis experienced by life on Earth, and the common paradigm persists that the biotic recovery from the extinction event was unusually slow and occurred in a step-wise manner, lasting up to eight to nine million years well into the early Middle Triassic (Anisian) in the oceans, and even longer in the terrestrial realm. Here we survey the global distribution and size spectra of Early Triassic and Anisian marine predatory vertebrates (fishes, amphibians and reptiles) to elucidate the height of trophic pyramids in the aftermath of the end-Permian event. The survey of body size was done by compiling maximum standard lengths for the bony fishes and some cartilaginous fishes, and total size (estimates) for the tetrapods. The distribution and size spectra of the latter are difficult to assess because of preservation artifacts and are thus mostly discussed qualitatively. The data nevertheless demonstrate that no significant size increase of predators is observable from the Early Triassic to the Anisian, as would be expected from the prolonged and stepwise trophic recovery model. The data further indicate that marine ecosystems characterized by multiple trophic levels existed from the earliest Early Triassic onwards. However, a major change in the taxonomic composition of predatory guilds occurred less than two million years after the end-Permian extinction event, in which a transition from fish/amphibian to fish/reptile-dominated higher trophic levels within ecosystems became apparent.

## Introduction

The evolution of life on earth can be broadly characterized by a continuum of periods of biodiversification, turnover events, and times of crisis where extinction occurred on a large scale, thus allowing us to study biotic and abiotic ecosystem fluctuations throughout the Phanerozoic, the most severe of which were centered around the end-Permian mass extinction [1]–[6]. To better understand the dynamics involved, it is necessary to consider and evaluate potential food webs directly after extinction events and during the following recovery phases. By occupying the top of the food webs, apex predators are highly susceptible to environmental fluctuations and stress [7], [8] and, therefore, they are key for understanding ecosystem recovery after extinction events (see below). However, limited research on Early Triassic top marine predators still obscures the pattern of recovery among higher trophic guilds after the largest mass extinction event in Earth's history near the Permian-Triassic (PT) boundary, about 252 million years ago [1], [9], [10].

Throughout the last decade, several papers were published that focused on a variety of southern Chinese Triassic biotas and sites of different geological ages, e.g., Chaohu (Anhui Province, late Olenekian, late Early Triassic), Panxian (Guizhou Province, middle Anisian, early Middle Triassic), Luoping (Yunnan Province, middle to late Anisian), Xingyi (Guizhou, late Ladinian, late Middle Triassic), and Guanling (Guizhou, early Carnian, early Late Triassic), yielding in many cases new taxa and well-preserved marine vertebrate fossils [11]–[17]. Two of these biota were subsequently used to infer the timing of the marine biotic recovery from the end-Permian mass extinction, proposing that full recovery was not reached until either in the middle Anisian as shown by the Luoping biota [11], [18], [19], or even later in the Late Triassic with the Guanling biota [15], [17].

A recent review article [20], whose aim was to summarize the factors and patterns involved in the biotic recovery from the end-Permian event, follows the previous interpretation that the middle to late Anisian fossil site of Luoping [18], [19] represents one of the earliest recovered ecosystems worldwide. The authors thus adhere to the conventional interpretation in which the recovery phase following the PT-boundary is prolonged for up to 8 million years into the Middle Triassic ([21], [22] and references therein). In reference to terrestrial ecosystem “disaster faunas”, it was pointed out that species evenness was also very low in the marine realm and that the trophic pyramid was rebuilt step-by-step throughout the Early Triassic and Anisian by adding new, higher levels [20]. Species evenness is one of the basic parameters of community structure, indicating the abundance of species coexisting in an ecosystem: high species evenness indicates species are evenly abundant, whereas low species evenness shows that some species are more abundant and thus, are dominant over others [23]. On p. 377 [20] it was further noted that in the Luoping biota “[…] the 25 species of fishes and diverse marine reptilians, comprising together 4% of finds, show multiple new predatory levels in the ecosystems […]”, but they do not explain which of those were supposedly missing in the Early Triassic.

Why is it important to examine the recovery patterns of apex predators (i.e., upper trophic level predators;  =  top predators) following the end-Permian mass extinction? Studies of modern ecosystem dynamics indicate the crucial role that apex predators play in stabilizing ecosystems, and that the depletion of this guild can cause severe instabilities and loss of biodiversity [7], [8]. Conversely, we hypothesize that if apex predators are recovered from a fossil site, their presence would indicate a certain diversity and length of trophic chains in the ancient ecosystems in question (see below). We therefore conducted a comprehensive study of available data for Early Triassic and Anisian larger marine vertebrates (Chondrichthyes, Osteichthyes, Tetrapoda). Our data base includes information on species richness (i.e., fishes: a count of species for which size data are known; reptiles: all species were considered) and body size of osteichthyan and chondrichthyan fishes, as well as secondary marine tetrapods, namely temnospondyl ‘amphibians’ (mainly trematosauroids) and reptiles (e.g., thalattosaurs, ichthyosaurs, sauropterygians). This study aims to elucidate the patterns of spatial and size distribution of key marine predators following the PT-boundary mass extinction as an indicator of the length of food chains or the number of trophic levels. Due to the limited knowledge about body size in Chondrichthyes (fossils are mostly restricted to isolated teeth, fin spines or denticles) their role as marine apex predators is, with some exceptions, qualitatively discussed herein. This group is comprehensively studied elsewhere [24].

Because a study of the biodiversity of secondary marine tetrapods during the Mesozoic [25] investigated the diversity patterns at the stage level but not at the sub-stage or higher resolved biostratigraphic levels (i.e., zones and subzones), these data therefore are only marginally useful herein. Previous evaluations of fish diversity across the Permian-Triassic boundary [26]–[28], which basically show an increase in diversity following the PT boundary crisis, are also only of limited use for the aim of our study. Although the presented analysis does not adequately assess trophic network complexities [29] for the Early Triassic marine realm, food chain lengths ending with large top-predators nevertheless imply at least stacks of underlying trophic levels (including primary producers, primary and secondary consumers and higher predatory levels) and thus, help to illuminate recovery patterns of marine ecosystems after the end-Permian mass extinction.

**Figure 1:**
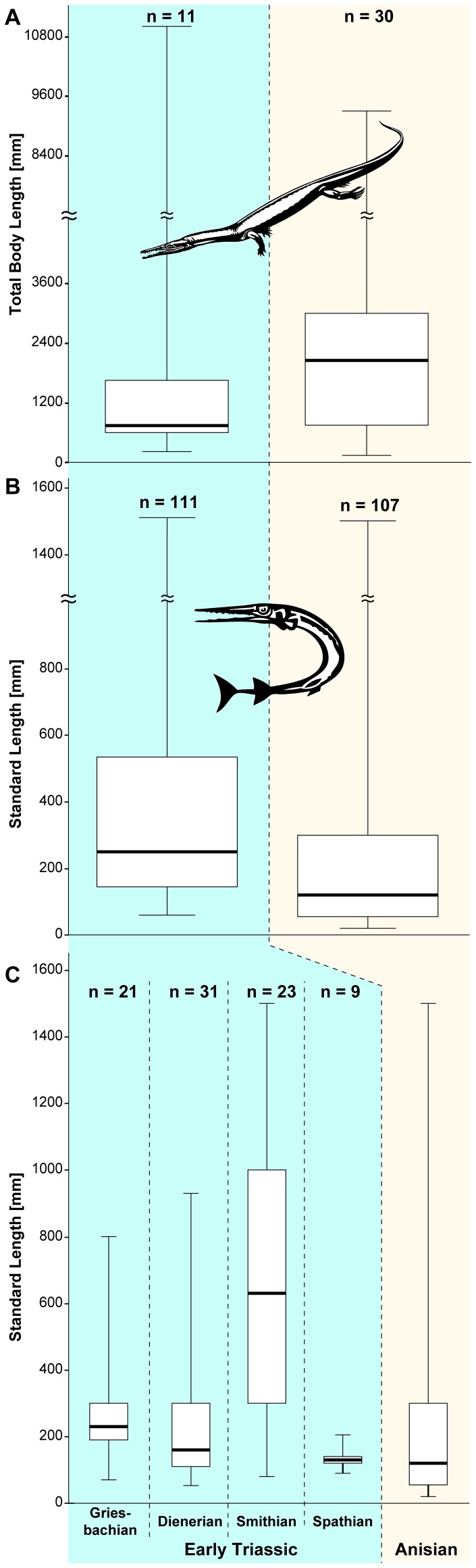
Boxplots showing maximum size ( =  total body length) of marine tetrapod (‘amphibians’, reptiles) and maximum standard lengths of marine non-tetrapod vertebrates (osteichthyans, chondrichthyians). A. Tetrapod data for the Early Triassic (11 taxa) and the Anisian (30 taxa). Note that the apparent increase in size is not significant. B, C. Non-tetrapod data comprising marine bony fishes (Actinistia, Actinopterygii) and some chondrichthyans with reliable body size estimates in the Early Triassic and the Anisian (early Middle Triassic). The upper two columns in (B) depict the pooled data, whereas in (C) the Early Triassic is split into the respective sub-stages. Based on data taken from the literature for 111 and 107 species for the Early Triassic and the Anisian respectively (see Table S1 in File S1). The boxes represent the 25–75 percent quartiles (bold horizontal lines indicate the medians) and the width of the tails the whole spread of data.

**Figure 2:**
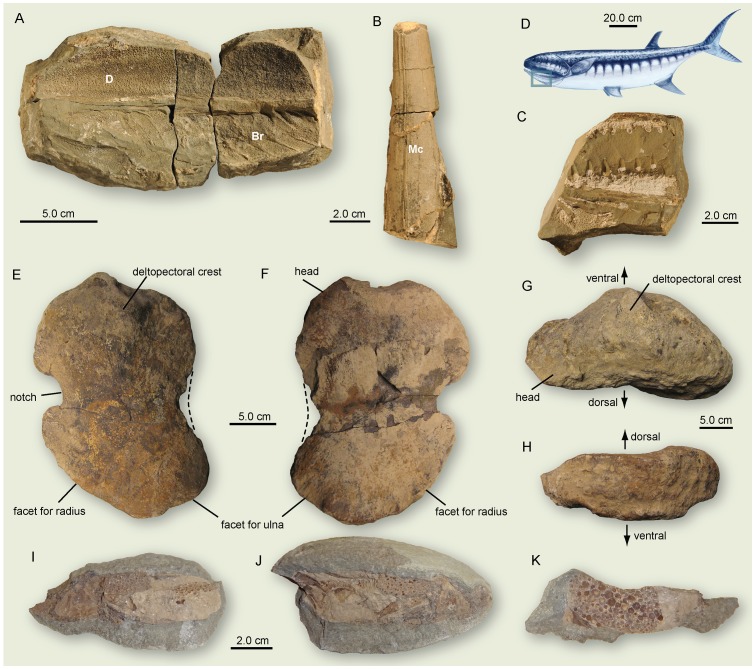
New fossil finds corroborating the presence of large predators in the Early Triassic. **A-C**. Assemblage of skull and lower jaw elements of a large *Birgeria* sp. (PIMUZ A/I 4301) from the Lusitaniadalen Member (Smithian), Vikinghøgda Formation, Stensiöfjellet, Sassendalen, Spitsbergen. Note that specimen (**B**) represents the infilling of the Meckelian canal. **D**. Position of the large specimen (**A**) on the reconstruction of animal indicated by blue rectangle. **E-H**. Humerus (NMMNH P-65886) of a giant ichthyosaur from the mid to late Spathian in the Hammond Creek area, Bear Lake valley, southeast Idaho, USA. **I-K**. Nodule (PIMUZ 30731) containing large coprolite with fish remains from the Griesbachian of Kap Stosch, East Greenland, possibly from a temnospondyl ‘amphibian’. Br, branchiostegal rays; D, dentary; Mc, Meckelian canal (infilling).

**Figure 3:**
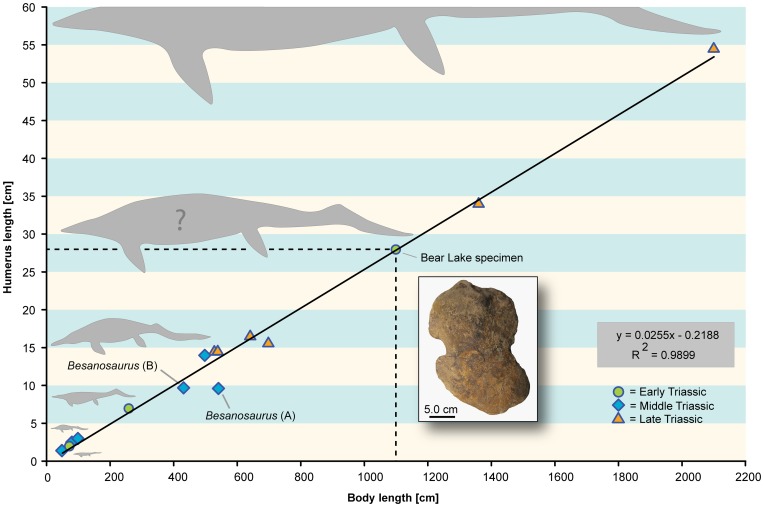
Humeral proximodistal length-body length relation in Triassic ichthyosaurs. Note that the upper two data points (*Shonisaurus popularis* and *Shastasaurus sikanniensis*) are based on estimated body lengths, whereas the other points rely on complete specimens. Removing the two taxa from the plot results in a shift of the specimen from Bear Lake (southwest Idaho, USA) towards even larger body size estimates.

Data for the present study are derived from the literature (Fig. 1; Table S1 in File S1), as well as new specimens (Figs. 2, 3). Species relative abundance (e.g. beta diversity), which would be a better measure of biodiversity than pure species counts [30], is more difficult to assess because, in many instances, species abundance has not been quantified, fossils are fragmentary and can only be assigned to higher level taxonomic clades, or the exact location of a particular fossil find is not well known. It is also noteworthy that in the last decade, the Early Triassic time scale has been increasingly refined using combined ammonoid and/or conodont faunas with radiometric dates, thus leading to re-definitions of Triassic stage and sub-stage boundary ages [9], [10], [31]–[34]. For example, in just six years, the Permian-Triassic boundary shifted from 251.0 Ma to 252.2 Ma and the Olenekian-Anisian boundary from 245.0 Ma to 247.2 Ma, respectively [10], [35]. Furthermore, index fossils (fossils considered to be characteristic of a certain time period only) are sometimes found to have diachronous first occurrences. Just such a case involved the supposed earliest Anisian-aged conodont *Chiosella timorensis*, a proposed index fossil for the Olenekian-Anisian boundary, which was recently shown to actually overlap in stratigraphic occurrence with Late Spathian ammonoids [36]. A similar case of diachronous first occurrence is also documented for the base of the Triassic with the index conodont species *Hindeodus parvus* [37]. These and other examples of course have implications for the accuracy of the timing of Early Triassic biotic recovery.

## Materials and Methods

### Institutional Abbreviations

BES, Paleontological collection of the Museum of Natural History of Milan, Italy; BSP, Bayerische Staatssammlung fur Paläontologie und Historische Geologie, Munich, Germany; CCCGS, Chengdu Center of China Geological Survey, Chengdu, China; CMC, Cincinnati Museum Center, Museum of Natural History and Science, Cincinnati, Ohio, USA; GMPKU, Geological Museum of Peking University, Beijing, China; GMR, Geological Survey of Guizhou Province, Guiyang, China; IVPP, Institute of Vertebrate Paleontology and Paleoanthropology, Beijing, China; MNHN, Muséum National d′Histoire Naturelle, Paris, France; NMNS, National Museum of Natural Science, Taichung, Taiwan; NMMNH, New Mexico Museum of Natural History and Science, Albuquerque, New Mexico, USA; PIMUZ, Paleontological Institute and Museum, University of Zurich, Zurich, Switzerland; SMNS, Staatliches Museum für Naturkunde, Stuttgart, Germany; TMP, Royal Tyrrell Museum, Drumheller, Alberta, Canada; UCMP, University of California Museum of Paleontology, Berkeley, California, USA; YIGMR, Wuhan Institute of Geology and Mineral Resources (former Yichang Institute of Geology and Mineral Resources), Hubei Province, China; YPM, Yale Peabody Museum, New Haven, Connecticut, USA; ZMNH, Zhejiang Museum of Natural History, Hangzhou, Zhejiang, China.

The fossil specimen (NMMNH P-65886, ichthyosaur humerus) is stored and curated at the New Mexico Museum of Natural History and Science, 1801 Mountain Road NW, Albuquerque, New Mexico, 87104, USA (NMMNH). It was originally collected on private property before it was donated to the museum, so no collection permits were necessary. The specimen was originally collected from the surface just above Horizon H19 of Guex *et al*. [38] at the Hammond Creek locality (Bear Lake County, SE Idaho), which contains only Spathian-aged marine sediments and ammonoids (*Ceccaisculitoides hammondi; Silberlingeria bearlakensis; Silberlingeria coronata; Silberlingeria sarahjanae*). The age of the humerus, although not found deeply embedded in the rock, is still well constrained based on the fact that a) ammonoids of latest Late Smithian age do not occur in Hammond Creek and b) marine Middle Triassic strata do not occur anywhere in Idaho. The closest marine strata of Middle Triassic age (Middle Anisian) are in central Nevada, over 300 miles further to the S-SW of the Hammond Creek locality.

Specimens PIMUZ 30731 (coprolite) and PIMUZ A/I 4301 (*Birgeria* sp.) are stored and curated at the Paläontologisches Institut und Museum, Universität Zürich, Karl Schmid-Strasse 4, CH-8006 Zürich, Switzerland. The former was collected with permission of the Aatsitassanik ikummatissanillu Pisortaqarfik, Råstofdirektoratet, Bureau of Minerarals and Petroleum, Government of Greenland (licence holder HB), and the latter with permission of the Sysselmannen på Svalbard, Longyearbyen, (Governor of Svalbard; application in collaboration with the Natural History Museum, University of Oslo, Norway). All necessary permits were obtained for the described study, which complied with all relevant regulations.

### Measurements

The maximum standard length (MSL) of marine species of bony fishes (Actinistia, Actinopterygii) and some cartilaginous fishes (Chondrichthyes) of Early Triassic and Anisian (Middle Triassic) age is mainly based on literature data (Fig. 1 and Table S1 in File S1). MSL of Triassic predatory fishes *Birgeria* and *Saurichthys* was in some cases estimated based on available material in comparison with more complete specimens. In general, the skull length of *Saurichthys* usually measures one fourth to one third of the standard length [39], [40]. In *Birgeria*, the skull (without pectoral girdle) usually makes up nearly one fifth of the standard length (cf. [41], [42]). Where a range is given in Table S1 in File S1, the mean value was used for the box plot analysis (Fig. 1). Where a minimum or maximum length is given (indicated by the > or < symbols), the appropriate number was used for the box plot analyses, assuming that these values approximately represent the size of the fish. With some exceptions (see Table S1 in File S1), MSL in chondrichthyans is difficult to estimate. In higher tetrapod clades (temnospondyl ‘amphibians’ and reptiles) diversity and size spectra are also difficult to assess because of preservational artifacts. Where appropriate, maximum length ( =  total size) was measured or estimated based either on the literature or on real specimens, whereas the remainder of the taxa were discussed qualitatively. Note that throughout the article, the term *amphibian* is used in quotation marks to indicate that we refer to extinct stem-amphibians herein and not to crown Lissamphibia.

Fossils were measured (Fig. 2, 3; Table S3 in File S1) with a band scale and calipers or digitally, using the software Fiji [43]. Statistical analyses were performed using the open access software PAST [44].

## Results

### The Marine Fish Record

Of the various groups of fishes and fish-like basal vertebrates, only four lineages cross the Permian-Triassic boundary: ‘Cyclostomata’ (hagfishes, lampreys and their fossil relatives; [45]), Conodonta (basal jawless animals with teeth-like elements and controversial systematic affinities; [46]–[48]), Chondrichthyes (cartilaginous fishes: sharks and their relatives [28]) and Osteichthyes (bony fishes: lungfishes, actinistians and actinopterygians [28], [49]). ‘Cyclostomata’, while generally rare in the fossil record, are not yet known from the Early Triassic. While conodonts undoubtedly represent a major component as both predators as well as prey items in the ancient ecosystems of Permian and Triassic times [50], it is only the cartilaginous and bony fishes (see below) that constituted the large predators among the non-tetrapod vertebrates in the marine realms at that time. Fishes, especially actinopterygians, generally exhibit an increase in diversity at the beginning of the Mesozoic, and reach their first peak in the Middle Triassic [26]–[28], [51], [52]. A specific radiation event has been recently proposed for neoselachian and hybodont chondrichthyans at the Permo-Triassic boundary, partly as a response to the extinction of previously abundant Palaeozoic stem chondrichthyans (e.g., Stethacanthidae) [53], although some clades such as cladodontomorph chondrichthyans might have survived into the Triassic utilizing deep-sea refugia [54].

In contrast to the southern Chinese localities mentioned above, the classical Early Triassic vertebrate sites in Greenland, Spitsbergen (Arctic Norway), Madagascar and British Columbia (Canada) are characterized by high abundances of fish fossils and diverse ichthyofaunas (e.g. [55]–[58]; CR & HB pers. obs.). Fishes from these sites exhibit a wide spectrum of shapes and sizes [59] ranging from more general fusiform species like *Boreosomus* Stensiö, 1921 and *Pteronisculus* White, 1933 ( =  *Glaucolepis* Stensiö, 1921) to deep-bodied forms such as *Bobasatrania* White, 1932, and the garfish-shaped predatory actinopterygian *Saurichthys* Agassiz, 1834.

Conversely, Early Triassic marine fishes from China are still very poorly known compared to those from the Middle Triassic of this region, and are basically restricted to Chaohu in Anhui Province, Jurong in Jiangsu Province, Zuodeng in Guangxi Province, and Changxing in Zhejiang Province [11], [59]. Most of these faunas are of Spathian age [60], [61] and include relatively small parasemionotid and “perleidid” actinopterygians, but *Saurichthys* and predatory hybodontoid sharks have also been mentioned ([60], [62]–[66]; see [59] for research history). Most of these faunas have only recently been studied and are still poorly understood. Hence, the Chinese record alone is not suitable for a discussion of global recovery patterns of fishes after the end-Permian mass extinction (*contra* [11], [15], [18], [20]).

We have compiled a record of the maximum standard length (MSL) of marine Actinistia and Actinopterygii (Osteichthyes) and some Chondrichthyes known by more complete fossil remains from the Early Triassic and Anisian (see Table S1 in File S1) Body size in fishes is a proxy for trophic level affiliation, as was recently demonstrated for extant taxa [67], [68]. Our results show that marine bony fishes occupied a similar spectrum of body size during the Early Triassic and the Anisian (Fig. 1), ranging from a few centimeters to at least 1.5 meters (Table S1 in File S1). However, in total median MSL of fishes was larger in the Early Triassic than in the Anisian (Mann-Whitney U test, p<0.01) [44]. Moreover, the distribution of MSL was also shifted towards larger body sizes in the Early Triassic compared to the Anisian (Kolmogorov-Smirnov test, p<0.01). Body size changes between the Early and Middle Triassic are also seen in some families, for instance, Middle Triassic bobasatraniids and actinistians attained MSLs of only a few tens of centimeters and were thus much smaller than some of their Early Triassic relatives that achieved body lengths greater than 1 meter (Table S1 in File S1). Our compiled data representing MSLs of fishes clearly contradict the claim that higher trophic levels were absent from marine ecosystems during the Early Triassic and, thus, refutes the stepwise recovery model of the trophic pyramid [20].

#### Chondrichthyes

**Figure 4:**
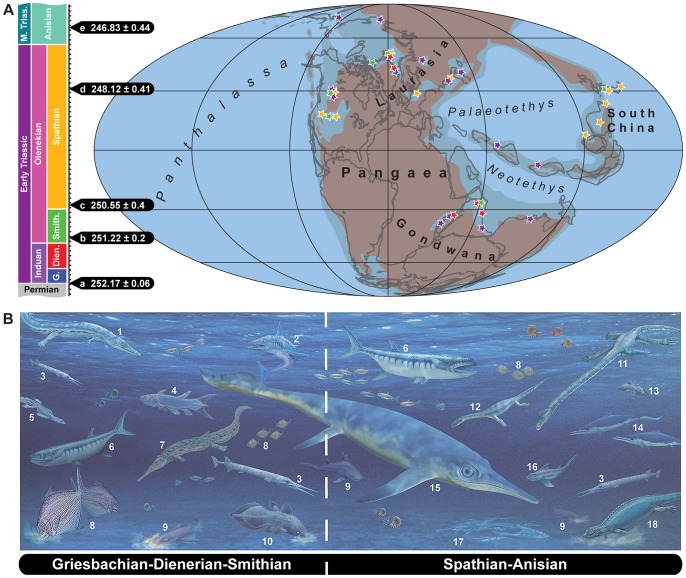
Spatial and stratigraphical distribution of Early Triassic and Anisian (early Middle Triassic) marine vertebrate predators. **A.** Geological time scale (Permian-Middle Triassic) with absolute time calibration according to radiometric UPb ages: a based on [9]; b on [34]; c–e on [33]. Paleogeographical distribution of selected marine predatory vertebrates is given on the right using the same color code as in the geological time scale (globe modified from C. Scotese’s paleomap project; http://www.scotese.com). **B.** Marine vertebrate apex predators during the Griesbachian to Smithian interval (left) and the Spathian to Anisian interval (right). Predators not exactly to scale; see text and Tables S1–S2 for details on body size and stratigraphic occurrence. Marine vertebrate apex predators: 1, *Wantzosaurus* (trematosaurid ‘amphibian’); 2, *Fadenia* (eugeneodontiform chondrichthyan); 3, *Saurichthys* (actinopterygian ambush predator); 4, *Rebellatrix* (fork-tailed actinistian); 5, *Hovasaurus* (‘younginiform’ diapsid reptile); 6, *Birgeria* (fast-swimming predatory actinopterygian); 7, *Aphaneramma* (trematosaurid ‘amphibian’); 8, *Bobasatrania* (durophagous actinopterygian); 9, hybodontoid chondrichthyan with durophagous (e.g. *Acrodus*, *Palaeobates*) or tearing-type dentition (e.g. *Hybodus*); 10, e.g., *Mylacanthus* (durophagous actinistian); 11, *Tanystropheus* (protorosaurian reptile); 12, *Corosaurus* (sauropterygian reptile); 13, e.g., *Ticinepomis* (actinistian); 14, *Mixosaurus* (small ichthyosaur); 15, large cymbospondylid/shastasaurid ichthyosaur; 16, neoselachian chondrichthyan; 17, *Omphalosaurus* skeleton (possible durophagous ichthyosaur); 18, *Placodus* (durophagous sauropterygian reptile). Printed under a CC BY license, with permission from Nadine Bösch and Beat Scheffold, original copyright [2013].

Cartilaginous fishes are usually represented in the fossil record as isolated teeth, dermal denticles, fin spines or cephalic spines. Due to the reduced fossilization potential of cartilage compared to apatite (e.g. bones and teeth), complete body fossils of chondrichthyans are rare. Therefore, data concerning body size of chondrichthyans are relatively sparse (Table S1 in File S1). However, chondrichthyan teeth are often abundant in micro- and macrofossil assemblages and they provide valuable information regarding the dimensions and diet of the animals to which they belonged. The Early Triassic record of Chondrichthyes includes not only predatory forms with tearing-type teeth (e.g., *Hybodus rapax* Stensiö, 1921, with teeth that are at least 23 mm long and 32 mm high), but also durophagous groups (e.g., *Acrodus* Agassiz, 1837, with teeth of up to 24 mm length: [58], [69]; *Palaeobates polaris* Stensiö, 1921, with teeth of up to 15 mm length and an estimated body length of ca. 100 cm: [70]). Other possible hybodontoids of Early Triassic age such as *Homalodontus* Mutter, Neuman & de Blanger, 2008 [71] ( =  *Wapitiodus* Mutter, de Blanger & Neuman, 2007) also reached large sizes of up to 150 cm [72]. Hybodontoids (*sensu* [73]), one of the dominant group of Mesozoic chondrichthyans, were already widespread at the onset of the Triassic [59]. Neoselachii, the clade that includes all extant chondrichthyans, have been known since the Paleozoic and are also occurring in Early Triassic fossil fish assemblages (e.g., [24], [74], [75]). Eugeneodontiformes (Fig. 4), a group of Paleozoic “tooth-whorl” bearing chondrichthyans that included such iconic forms as *Helicoprion* Karpinsky, 1899 from the Permian [76], exhibits various tooth morphologies and has its last occurrence in the Early Triassic [24], [77], [78]. This enigmatic group comprises Early Triassic species ranging from 100 to 150 cm in length (e.g. *Caseodus* Zangerl, 1981, *Fadenia* Nielsen, 1932), similar in size to their Paleozoic relatives [78]. *Fadenia*, for instance, possessed a large, homocercal caudal fin [78] that is typical for fast-swimming, active predators. Eugeneodontiform teeth have been recovered from various world-wide Early Triassic deposits, including western Canada [78], Spitsbergen [79], Greenland [80], Azerbaijan [81], [82] and South Tibet [83], thus demonstrating the widespread existence of the group prior to its extinction in the late Early Triassic [24], [77].

Another Paleozoic survivor genus is *Listracanthus* Newberry & Worthen, 1870, a chondrichthyan of unknown systematic affinities. This taxon has been described from the Early Triassic of western Canada [56], [84], from strata of Smithian or older age. As for the Eugeneodontiformes, *Listracanthus* disappears from the fossil record in the Early Triassic. Although *Listracanthus* is only known from denticles, it was suggested to be of large size and, hence, would classify as yet another chondrichthyan predator of Early Triassic age [84]. Mutter & Neumann [85] speculated that the large denticles of *Listracanthus* could represent gill rakers of a large filter-feeder. However, besides this dubious case, there is no fossil evidence for filter-feeding fishes or tetrapods in the Early Triassic. Furthermore, a lilliput effect was proposed for *Listracanthus* based on changes in denticle size during the Early Triassic [85] in comparison to the older records of the taxon. However, this interpretation seems questionable as changes in size of denticles do not necessarily reflect differences in body size [28].

#### Osteichthyes

Early and Middle Triassic marine bony fishes include actinopterygians (ray-finned fishes) and actinistians (coelacanths). Dipnoans (lungfishes) were restricted to the freshwater realm (apart from a few possible exceptions; [86]) and are therefore not considered herein. Compared to Chondrichthyes, the potential for fossilization of Osteichthyes is generally higher. Marine bony fishes exhibit an overwhelming diversity of body shapes and sizes during the Early Triassic, including small to mid-sized fusiform taxa (e.g. *Boreosomus*, *Pteronisculus*, *Helmolepis* Stensiö, 1932; Parasemionotidae: [55], [57], [58], [63], [64]), small to very large deep-bodied forms (e.g. *Bobasatrania*, *Ecrinesomus* Woodward, 1910: [56], [57], [87], [88]), as well as large fast-swimming predators (*Birgeria* Stensiö, 1919, *Rebellatrix* Wendruff & Wilson, 2012) and small to large ambush predators (*Saurichthys*: [89], [90]). It has been shown that many genera achieved a global distribution during the Early Triassic [24], [56], [59], [91].

Actinopterygians, which make up the bulk of bony fishes, had already developed different feeding specializations in the earliest Triassic (Griesbachian). This group includes small to large durophagous forms (Fig. 4; e.g. *Bobasatrania* with pharyngeal tooth plates: [51], [57]), as well as mid-sized (e.g., *Pteronisculus*: [55], [92]) and large carnivores (Fig. 4; e.g. *Birgeria*, *Saurichthys*: [41], [89]). The latter two taxa, *Birgeria* (Fig. 2A-C) and *Saurichthys*, the piscine apex predators of the Triassic [93], retained the same maximum body size of ca. 1.5 meters during the Early and Middle Triassic ([42], [58], [90], [94], [95] HB & JJ pers. obs.). Other marine Early Triassic fishes such as parasemionotids and platysiagids, both of which are known from various paleogeographic regions, remained relatively small (normally below 20 cm) as adults and, thus, would represent lower trophic levels [55], [57], [96].

*Birgeria* and *Saurichthys* are known at least from the Griesbachian onwards [57], [89], and both taxa exhibit a cosmopolitan distribution during the Early Triassic [42], [97]. Although *Saurichthys* fossils are relatively sparse in the Early Triassic of East Greenland (14 specimens [89]), remains of *Birgeria* are quite common (107 specimens [41]) in this region. *Birgeria* and *Saurichthys* are also known from abundant material from the Early Triassic of the USA and Canada (ca. 52 specimens of *Saurichthys*: [56], [95], [97]; *Birgeria* is rare) and Spitsbergen (59 specimens of *Saurichthys*: [90], and nine of *Birgeria*: [58], [90]). Although only a few specimens of *Birgeria* and *Saurichthys* from Madagascar have so far been mentioned in publications [55], [98]–[102], well over 100 additional yet undescribed individuals of *Saurichthys* are distributed in museum collections (e.g. Paris, Freiberg, Zurich; CR pers obs., I. Kogan pers comm. to CR). Taking sampling bias (see below) into account, these numbers are comparable with the Middle Triassic record: e.g. at least 67 specimens of *Birgeria* and about 320 individuals of *Saurichthys* from Monte San Giorgio area, southern Switzerland and northern Italy [40], [42], and more than 150 specimens of *Saurichthys* (including *Sinosaurichthys* Wu, Sun, Hao, Jiang, Xu, Sun & Tintori, 2011) from southern China [103]–[105]. Hence, at the global scale *Birgeria* and *Saurichthys* cannot be considered rare in the Early Triassic (contra [20]: p. 379).

Actinistians, which show generally low diversity in the fossil record, achieved their all-time highest diversity during the Early Triassic, with at least 13 valid genera ([97], [106] and references therein). This group includes small to mid-sized taxa (e.g. *Piveteauia* Lehman, 1952: [55], [107], *Chaohuichthys* Tong, Zhou, Erwin, Zou & Zhao, 2006 [64], *Belemnocerca* Wendruff & Wilson, 2013 [106]), as well as very large forms. For example, a body length of 600–800 mm has been estimated for *Mylacanthus* Stensiö, 1921 and *Wimania* Stensiö, 1921 from the Smithian of Spitsbergen ([58]) and the recently discovered *Rebellatrix* (Fig. 4) from the Early Triassic of western Canada reached an estimated length of 1300 mm [108]. Some Early Triassic actinistians were fast-swimming predators (*Rebellatrix*), while others (e.g., *Axelia, Mylacanthus*) had a slow-moving benthos-oriented lifestyle and a durophagous diet ([57], [58], [108]).

### The Marine Tetrapod Record

Among Tetrapoda, temnospondyl ‘amphibians’, procolophonid parareptiles, eutherapsid synapsids (anomodonts and eutheriodonts, the latter leading to modern mammals), and basal eureptiles (which later gave rise to modern reptile groups like lizards, snakes and crocodylians) survived the Permian-Triassic extinction event [5], [109]–[115]. Of these groups, only the temnospondyl ‘amphibians’ and eureptiles (e.g. ‘younginiform’ eureptiles, protorosaurian archosauromorphs) include species that are adapted to marine life.

Previous studies [116]–[118] have already noted that the marine reptile diversity seen in the Spathian implies an even older, as yet unrecognized evolutionary record from ancestors with an amphibious lifestyle, and they also pointed out the importance of reptiles for Mesozoic marine ecosystems. These studies, however, were published either before or they did not take into account the latest taxonomic descriptions and revisions of marine reptiles available to date [119]–[122]. A recent summary of Chinese Triassic marine biota indeed similarly implies early diversification of marine reptiles throughout much of the Early Triassic and even close to the Permian-Triassic boundary [11]. Walker & Brett [123] further summarized patterns of predation both among marine vertebrates and invertebrates thoughout large parts of the Phanerozoic. An overview of Triassic marine reptiles was recently provided [124], in which the authors note that rates of sea-level changes may have been an important factor influencing nearshore marine ecosystems and thus the evolution and selective extinction of secondary marine reptiles during the Triassic. In this respect, it is worth noting that the Smithian-Spathian boundary has long been recognized as a regression peak [125].

The oldest remains of Tanystropheidae, a group of protorosaurian archosauromorphs that included iconic forms such as *Tanystropheus* Meyer, 1852 (Fig. 4) with extremely elongated neck vertebrae [126]–[128], were recently described from the late Early Triassic of the Volgograd Region, western Russia [129], thus constituting yet another, highly specialized predator in the Early Triassic. Whether Hupehsuchia, a group of diapsid reptiles that may be closely related to ichthyosaurs [130], is present as well in the Early Triassic remains under discussion (see below). Of the marine reptile groups studied, the ichthyosaur fossil record in particular yielded many large to enormously large but disarticulated body fossils of late Early Triassic age, as demonstrated for example by the discovery of a giant humerus from the mid-late Spathian of the Thaynes Formation (Idaho, western USA, Figs. 2D-G, 3). This occurrence supports the hypothesis that ichthyopterygians experienced a burst of diversification and adaptive radiation [131] well before the Middle Triassic. A list of tetrapod species surveyed herein is presented in Table S2 in File S1.

#### Temnospondyl ‘Amphibians’ (Mainly Trematosauroidea)

Stereospondyli are a widespread group highly nested within temnospondyl ‘amphibians’, which are known to occur from the Late Permian to Early Cretaceous. Whereas most stereospondyls probably inhabited freshwater lake and river habitats, the trematosauroids are the lineage of temnospondyls considered to have been most successful entering the marine realm ([132], [133], often showing extremely elongated gharial-like skulls with numerous pointed teeth, implying a piscivorous diet [134]–[137]). Most trematosauroids were small to medium-sized predators ranging between one and two meters of total body length [122]. The skull of *Wantzosaurus elongatus* Lehman, 1961 ([137]: Fig. 4B) could reach 40 cm in length.

Specimens of trematosauroids, e.g., *Aphaneramma kokeni* Welles, 1993 [138], from the Salt Range of Pakistan are already known from the “*Prionolobus* beds”, Mittiwali Member, Mianwali Formation at Chiddru (e.g., [139], [140]), previously identified as either Griesbachian [141] or latest Dienerian [138]. Based on the newest detailed work on ammonoid faunas from the Salt Range, the so-called “*Prionolobus* beds” ( =  Ceratite Marls) at Chiddru are actually early Smithian in age [6] (*Wantzosaurus* Lehman, 1955, on the other hand, is known from older (Griesbachian?) sediments of Madagascar (Fig. 4A; [122], [138]). A rich temnospondyl fauna (an ‘amphibian’ fauna first studied in the 1930s by Säve-Söderbergh [142], who noted 40 specimens) from the ‘Stegocephalian zone’ and underlying ‘fish zones’ [143] of the Wordie Creek Formation of central East Greenland was reported [144], which is most likely Dienerian in age ([145], [146], HB pers. obs.). However, the taxonomic status of many of the originally described Early Triassic species from Greenland largely remains obscure [137], [144]. Three genera from the Wordie Creek Formation at Kap Stosch in East Greenland are considered valid ([122], [144], see also [147]): the capitosauroid *Aquiloniferus* Bjerring, 1999 (based on material that was previously referred to species of *Luzocephalus* Shishkin, 1980), the trematosaurid *Stoschiosaurus* Säve-Söderbergh, 1935 and the wetlugasaurid trematosauroid *Wetlugasaurus* Riabinin, 1930.

Research on the temnospondyl fauna of Spitsbergen preceded that of Greenland, starting with the works of Carl Wiman [133], [148], [149]. In addition to the non-trematosauroid basal stereospondyl *Peltostega* Wiman, 1916 (Rhytidostea), at least five trematosauroid genera are recognized in the ‘Fish Niveau’ (Lusitaniadalen Member) of the Vikinghøgda Formation ( =  Sticky Keep Formation) of Spitsbergen (Smithian, [150]), namely the trematosaurids *Aphaneramma* Woodward, 1904, *Lyrocephaliscus* Kuhn, 1961 ( =  *Lyrocephalus* Wiman, 1913), *Platystega* Wiman, 1914, and *Tertrema* Wiman, 1914, as well as the?wetlugasaurid *Sassenisaurus* Nilsson, 1942 [122], [133], [151], [152]. It is noteworthy that the latter four taxa exhibit shorter, more triangular cranial shapes as opposed to the extremely longirostrine skull of *Aphaneramma* (Fig. 4).

Recently published data [153] demonstrated that both trematosaurid subgroups, the shorter-snouted Trematosaurinae and the longer-snouted, gharial-like Lonchorhynchinae, were already present in the earliest Triassic (Griesbachian) and that trematosauroids had already achieved global distribution by that time [122], [154]. Hammer [154] hypothesized that trematosaurids are euryhaline predatory animals that preferred nearshore marine to distal deltaic habitats, based on the associated invertebrate faunal elements such as ammonoids and bivalves. A recent study concerning the bite-forces of temnospondyls further corroborated that within the trematosaurids, long-snouted forms such as *Wantzosaurus* and *Aphaneramma* were fully aquatic and preyed upon fast animals such as small fishes [155], and in the case of the former, a pelagic lifestyle has even been proposed [137]. Dwellers preferring a more coastal/near shore marine habitat may be represented by *Erythrobatrachus* Cosgriff & Garbutt, 1972 from the Upper Blina Shale (Olenekian) of West Kimberley, Western Australia, and *Cosgriffius* Welles, 1993 from the Wupatki Member, Moenkopi Formation (Spathian) of Arizona, USA [122]. It is noteworthy here that pelagic trematosauroids are known only from the Griesbachian to Smithian interval, and their almost complete disappearance from the fossil record roughly coincides with the first stratigraphic appearance of ichthyosaurs and sauropterygians (see below).

Furthermore, a whole range of non-trematosauroid temnospondyls is known from the Early Triassic of northwestern Madagascar [156], [157]. These animals are thought to be euryhaline, and at least the capitosaur *Edingerella madagascariensis* (Lehman, 1961) from the Ankitokazo Basin is thought to have also dwelled in brackish to costal, shallow marine habitats [157]–[159]. Bone histology of the armour elements of other non-trematosauroid temnospondyls underscores the ability of temnospondyls to tolerate changes in salinity and thus the assumption that they may have entered brackish or near-coastal marine habitats at least during short term hunts, even if they are not considered fully marine [160].

#### Sauropterygia

Sauropterygia, the widely distributed, diverse group of marine diapsid reptiles that gave rise to the Jurassic-Cretaceous plesiosaurs and pliosaurs is first reported from the Early Triassic. The earliest occurrence of the group is documented by the European record of non-diagnostic sauropterygian remains (Sauropterygia indet.), as well as *Corosaurus alcovensis* Case, 1936 from the Alcova Sandstone of Wyoming, USA [121], [161], [162]. Accordingly [121], [163], the earliest sauropterygian remains from both Europe and North America are of Spathian age ( =  late Early Triassic; in the Germanic Triassic: lower Röt Formation, upper Buntsandstein). Slightly younger remains referable to *Cymatosaurus* Fritsch, 1894 and *Dactylosaurus* Gürich, 1884 are known from the upper Röt Formation and the lower Muschelkalk of the Germanic Triassic, which is Aegean (early Anisian, Middle Triassic) in age.

Another early sauropterygian is the poorly known *Kwangsisaurus orientalis* Young, 1959 from the “[…] Loulou Group of the Beisi Formation upper Lower Triassic (lower Middle Triassic by some estimates)” of Guangxi Province, China ([164]: p. 325). The Early Triassic Luolou Formation is a mixed carbonate-siliciclastic formation deposited on the outer platform indicating moderately deep water settings, whereas the Beisi Formation is composed of limy mudstones, massive oolite grainstones and dolostone deposits in a shallow marine setting [165], [166]. The sauropterygians *Hanosaurus hupehensis* Young, 1972 (note that in the analyses of [167] *Hanosaurus* was recovered outside of Sauropterygia) and *Keichousaurus yuananensis* Young, 1965, together with hupesuchian remains (see below), have been reported from the Jialingjiang Formation of Wangchenkang (Yuan'an County, Hubei Province [168]), but the age of these fossils is still debated (either late Early Triassic or early Middle Triassic; [169], [170]). Accurate dating of these fossils is further complicated due to problems of the ill-defined Olenekian-Anisian boundary by means of conodont datums [36] (see also above).

With regard to the holotype of *C. alcovensis*, Storrs [171] provided a reconstructed skull length of slightly less than 15 cm and an estimated overall body length of 1.65 m. Isolated material (e.g. humerus YPM 41032) referable to *Corosaurus*, suggests the presence of larger individuals more than 4 meters in total length, thus exceeding most of the Middle and Late Triassic non-pistosauroid sauropterygians, including the huge predator *Nothosaurus giganteus* Münster, 1834 from the Germanic Muschelkalk sea and Alpine region [121] (‘*Paranothosaurus amsleri*’, a junior synonym of *N. giganteus*, from the UNESCO World Heritage Site of Monte San Giorgio, Switzerland, has an estimated body length of 3.85 m; [172]).

The durophagous placodonts (Fig. 4) are a specialized Triassic group of sauropterygians that includes both non-armoured and armoured species, of which the latter superficially resembles turtles (e.g., [173], [174]). Even though the earliest placodont fossils (i.e., *Placodus* Agassiz, 1833) were recovered from early Anisian sediments [121], [161], it is assumed that their evolutionary history reaches back into the late Early Triassic due to the high degree of aquatic adaptation noted in the earliest representatives of this clade [175]. Recently, new placodontiform reptiles [167], [176] were described from the lower Muschelkalk (Vossenveld Formation, early Anisian) quarry of Winterswijk, The Netherlands, including a tiny skull of a juvenile sauropterygian reptile, *Palatodonta bleekeri*, which in a phylogenetic analysis [167] was recovered as the direct sister taxon to Placodontia. It shared characters such as a single row of palatine teeth with Placodontia, but lacked any form of crushing dentition. Its basal morphology and place of recovery further argue for an origination of the group in Europe. The highly modified crushing dentition of placodonts [123], [177]–[180], as well as that of other durophagous marine vertebrates in the Early and Middle Triassic, indicates the importance of the crushing guild in the food web; also the diversity of these groups provides a proxy for the rate of sea-level changes during the Triassic period [124].

The discovery of new sauropterygian remains, besides possibly more basal diapsid remains, in the Middle to Upper Member of the Nanlinghu Formation in Majiashan, Chaohu, Anhui Province, southern China [169], [170], underscore the fact that the early evolution of the Sauropterygia as a whole is still largely obscured and that its origin definitely dates well back into the Early Triassic. These new sauropterygian fossils, which are contemporaneous with the ichthyopterygian *Chaohusaurus geishanensis* Young & Dong, 1972 (see below), argue directly and indirectly for the presence of placodonts in the Early Triassic, based on skeletal affinities to other known placodonts and through ghost lineage inference.

#### Thalattosauriformes

Thalattosaurs are a less diverse group of small to medium-sized (generally less than 4 meters in length) secondary marine reptiles restricted to the Triassic (Müller, Renesto & Evans, 2005). The oldest record of the group comes from the name-giving genus, *Thalattosaurus*, whose type species, *T. alexandrae* Merriam, 1904, was first described from the Carnian *Trachyceras* beds of the Hosselkus Limestone of Shasta County, California, USA [181]. Nearly one century later, newly discovered *Thalattosaurus* material from the Lower to Middle Triassic Sulphur Mountain Formation, Wapiti Lake area, British Columbia, Canada, was described as *T. borealis* [182], [183]. Material representative of two other taxa, *Paralonectes merriami* Nicholls & Brinkman, 1993 and *Agkistrognathus campbelli* Nicholls & Brinkman, 1993, was also recovered from the Sulphur Mountain Formation in British Columbia [183], [184]. All three taxa belong to the monophyletic clade Thalattosauridae within Thalattosauria (Thalattosauria and Askeptosauroidea are then combined into Thalattosauriformes: [185], [186]). Because many of the Canadian specimens were discovered as float in loose scree material on steep slopes [183], it is not possible to assign an exact age to these particular fossils. Nevertheless, specimens of *Paralonectes* and *Agkistrognathus* were found in a location (“cirque D”) where the sedimentary sequence extends from the Olenekian to the Middle Triassic [183].

#### Ichthyopterygia

Although the record of early ichthyosaurs is still very limited, it is apparent that at least since the Spathian (late Olenekian, late Early Triassic, Fig. 4A), the Ichthyopterygia as a group had already diversified and achieved global distribution, as revealed by discoveries from Asia, North America and northern Europe [119], [120], [187]–[193].

McGowan & Motani [120] recognized five well-known Early Triassic species of non-ichthyosaurian ichthyopterygians, namely *Chaohusaurus geishanensis* Young & Dong, 1972, *Grippia longirostris* Wiman, 1929, *Parvinatator wapitiensis* Nicholls & Brinkman, 1995, *Thaisaurus chonglakmanii* Mazin, Suteethorn, Buffetaut, Jaeger & Helmcke-Ingavat, 1991, and *Utatsusaurus hataii* Shikama, Kamei & Murata, 1978 ([194]–[198]). In addition, another grippidian ichthyosaur, *Gulosaurus helmi*, was recently described [199] based on material from the Vega-Phroso Siltstone Member (Early Triassic), Sulphur Mountain Formation, British Columbia, previously identified either as belonging to *Grippia* cf. *G. longirostris* [200] or to a juvenile specimen of *Parvinatator* [201].

Following the most recent work [202], *Omphalosaurus* Merriam, 1906 (Fig. 4), a durophagous marine reptile with a peculiar crushing dentition consisting of hundreds of stacked, bulbous teeth [202], can also be included within non-ichthyosaurian ichthyopterygians. Of these six taxa, only *C. geishanensis*, *G. longirostris* and *U. hataii* can be accurately dated (late Early Triassic), based on conodont and/or ammonoid age control [120], whereas *Omphalosaurus* is known from the Spathian to the early Ladinian (late Middle Triassic [202]). In the other cases the age control was rather loose and often the remains were found as surface float in assemblages of mixed ages.

*P. wapitiensis* is known from the Lower to possibly Middle Triassic Sulphur Mountain Formation of Wapiti Lake region, east central British Columbia (Canada) but fossils are usually recovered from loose slabs without further age control [120], [195].

*G. longirostris* was found in the “*Grippia* niveau”, the lower of the two tetrapod-bearing horizons in the upper Vikinghøgda Formation of Spitsbergen [203]. Both horizons belong to the latest Spathian *Keyserlingites subrobustus* Zone ([204] [120], which corresponds to the upper part of the Vendomdalen Member of the Vikinghøgda Formation, Sassendalen Group (*sensu* [205]). Several similarities in the dentition of *G. longirostris*, whose dentition was referred to the crunch guild [206], and *U. hataii* from Japan have been pointed out [207], [208]. A revision of the Svalbard ichthyopterygian fauna was recently provided [209] (also see below).

*U. hataii* is known from the Spathian Osawa Formation of Miyagi, Japan [120], [210], and material from the Sulphur Mountain Formation may also be referable to this genus [211]. This taxon was originally described from two profiles (Tatezaki and Osawa) in the Osawa Formation [196]. Dating of these fossils remains difficult, however. Only the upper *Utatsusaurus* occurrences in the profiles can unambiguously be correlated with the *Subcolumbites* Zone (e.g. through the occurrence of *Subcolumbites perrinismithi*, *Stacheites* sp., etc.). As for the findings in the lower part of the Tatezaki profile, the ammonoid correlation is incorrect, because, for instance, the older *Columbites parisianus* is mutually exclusive with the younger *Subcolumbites* or *Stacheites* [38]. The faults shown in the Tatezaki profile may further indicate repetition of sedimentary stacks, so that several specimens (A–D and L in [196]) cannot be precisely dated. The oldest, well-dated *Utatsusaurus* material thus appears to be restricted to the middle Spathian (*Subcolumbites* Zone; [212]), which makes it slightly older than the previously assumed late Spathian age [120].

*C. geishanensis*, one of the smallest forms, was found in the Spathian Qinglong Formation (*Neospathodus triangularis* conodont Zone and *Subcolombites* Zones) of Anhui, China [213], [214]. Its total body length is usually less than 1 meter, and it is regarded as one of the taxa unifying most of the plesiomorphic traits among ichthyosaurs [214]. New material of *Chaohusaurus* (more than eighty specimens by now) has recently been excavated from the Middle to Upper Member of the Nanlinghu Formation (Spathian) in Majiashan, Chaohu, Anhui Province [169], [170]. In addition, a new species of *Chaohusaurus*, *C. zhangjiawanensis*, was only recently described from the Jialingjiang Formation (*Neospathodus homeri*-*N. triangularis* conodont zone) of Yuanan, Hubei Province in South China [215]; the same formation that also yielded for example material of Hupesuchia (see below).

*Omphalosaurus* (type species: *O. nevadanus*), ([119], [202], [216], [217] was originally described from the late Early and Middle Triassic Prida Formation (Fossil Hill Member), Humboldt Range, Nevada, USA [190], [218]. Associated cranial and postcranial remains of *Omphalosaurus* cf. *O. nevadanus* (originally described as *O. wolfi*: [219]) are now known from the Middle Triassic (earliest Ladinian) of the Salzburg Alps, Austria, close to the German border [202], [220] and it showed affinities of the taxon to ichthyosaurs (but see [120] for different interpretation). Its lower jaw, which may have carried hundreds of rounded crushing teeth of various sizes, would exceed 50 cm in length if reconstructed [202]. The earliest records of this durophagous animal were described as *O. nettarhynchus* [190] already from the late Spathian (*Neopopanoceras haugi* Zone, Lower Member of the Prida Formation [218] of the Humboldt Range.

In addition to these better known basal ichthyopterygian species, representatives of more derived groups (e.g., mixosaurs, shastasaurids) which later flourished in the Middle and Late Triassic are also fragmentarily known from the late Early Triassic [120], [189], [221]. The shastasaurids in particular include large to giant-sized ichthyosaurs.

None of these early ichthyopterygians show the characteristic parvipelvian (meaning with a “small pelvis”) body shape of later ichthyosaurs, but instead had an elongate lizard-like body shape, an anguilliform (eel-like) mode of swimming, and they did not exceed three meters in total body size, with most species being smaller than one meter [214], [222].

The heterodont dentition found in many Early and Middle Triassic non-ichthyosaurian and ichthyosaurian ichthyopterygians (e.g., mixosaurs: [223], *Grippia*: [208]) may be indicative of a more omnivorous diet in these taxa [224]. A recent phylogenetic study indicates that *Xinminosaurus* Jiang, Motani, Hao, Schmitz, Rieppel, Sun & Sun, 2008, a form with crushing dentition from the Upper Member of the Guanling Formation at Panxian, Guizhou Province, China [225], might have had a ghost-range into the late Early Triassic as well [193].

The diverse ichthyopterygian fauna of the Svalbard archipelago was recently reviewed by Maxwell & Kear [209], who recognized six valid genera: *Grippia* Wiman, 1929, *Quasianosteosaurus* Maisch & Matzke, 2003, *Pessopteryx* Wiman, 1910 [226], *Omphalosaurus* Merriam, 1906, *Isfjordosaurus* Motani, 1999 and an additional indeterminate ichthyopterygian. *Isfjordosaurus minor* (Wiman, 1910) [226] and species of *Pessopteryx* Wiman, 1910 (including “*Rotundopteryx*” *hulkei* Maisch & Matzke, 2000 *sensu* [120]), which are also based on limited postcranial material from the “lower saurian niveau” (Vendomdalen Member) of the upper Vikinghøgda Formation of Spitsbergen, were treated as *species inquirendae* and *nomina dubia* respectively [120]. Later, a new genus name *Merriamosaurus* Maisch & Matzke, 2002 was introduced based on material of “*Rotundopteryx*” *hulkei* from the MNHN collections in Paris, France, which led to recognition and description of associated postcranial remains of a single individual among the otherwise isolated material [227], [228]. The taxon was described as a large species, which occupies “[…] the most basal position in Merriamosauria, forming the sister-group to *Besanosaurus* and all other more highly derived merriamosaurs” ([228]: p. 133). Following Maisch [188] and Maxwell & Kear [209], *Merriamosaurus hulkei* is herein treated as a junior synonym of *Pessopteryx nisseri* Wiman, 1910, a large ichthyosaur with shastasaurid affinities.

Furthermore, large teeth (more than 40 mm in length) from nodules at the base of the Spathian “*Grippia* niveau” were described as *Svalbardosaurus crassidens* [229], [230]. According to Maxwell & Kear [209], these teeth belonged to a large ‘amphibian’ rather than an ichthyosaur. Regardless of their systematic interpretation, these teeth still indicate the presence of very large predator in the Early Triassic of Spitsbergen. On the other hand, *Omphalosaurus*-like teeth were found among the material originally assigned to *Pessopteryx*. *Quasianosteosaurus vikinghoegdai*, an ichthyosaur whose skull length at least reached 50 cm, was also described from the lowermost “*Grippia* niveau” of the Vikinghøgda Formation [131]. According to these authors, *Quasianosteosaurus* anatomy compares closely to that of *Parvinatator*, with both classified as non-ichthyosaurian ichthyopterygians.

Recently, still undescribed ichthyosaur fauna was reported from the Fossil Hill locality, Prida Formation, of Pershing County, Nevada [231]. The discoveries were derived from Spathian-aged layers, similar to those from which *O. nettarhynchus* was described [190]. Preliminary tooth morphology studies indicate that an “*Utatsusaurus*-like form, and a *Chaohusaurus*/*Grippia*-like form” ([231]: p. 120) are among the new fossils. These taxa are more or less well-known from Canada, China, Japan, and Spitsbergen.

Material that was thought to represent *Cymbospondylus* Leidy, 1868 (several vertebrae, a distal portion of a left humerus, and some indeterminate bone) were described from the Olenekian Thaynes Formation at Nounan Valley, west of Georgetown, Bear Lake County, Idaho ([232], [233]; note that the morphological characters thought to be diagnostic for the genus by Massare and Callaway are not valid anymore, see [120]). As surface float collections within limestone nodules, these bones derived from the shales below the Platy Siltstone Member [232] from sediments of either Smithian or early Spathian age (see also [233]).

A new, unusually large ichthyosaur humerus (NMMNH P-65886, Fig. 2D-G) about 28.0 cm in proximodistal length that was also recently recovered from the mid-late Spathian portion (*Subcolumbites* Zone, biochronologic horizon 18 of [38]) of the Thaynes Formation (Hammond Creek, Bear Lake County, Idaho, USA), rivals the dimensions of some of the largest ichthyosaurs known. It is exceeded only by shastasaurids such as *Shonisaurus popularis* Camp, 1976 (maximum humeral length 43 cm; [234]) from the upper Carnian (Late Triassic) of Nevada, USA, and the gigantic *Shastasaurus sikanniensis* (nov. comb. by [235]; originally described as *Shonisaurus sikanniensis* in [234]) from the Late Triassic of British Columbia, which is estimated to have reached up to 21 m in total body length and whose humerus measured an amazing 54.5 cm in proximodistal length [234]. A highly predatory species, *Thalattoarchon saurophagis*, with flattened cutting-edged teeth from marine sediments of Middle Triassic age, Augusta Mountains, Pershing County, Nevada, was also estimated to have reached more than 8.6 meters in body length [236]. The new humerus from Hammond Creek resembles the deeply notched humeri of certain Late Triassic ichthyosaurs, fitting humerus morphotype 2 *sensu* [237], which is characteristic for Shastasauridae Merriam, 1902. It most closely resembles the deeply notched humerus of *Callawayia neoscapularis* (McGowan, 1994) from the Late Triassic Pardonet Formation of British Columbia ([119], [120], see also [237], [238]) or *Shastasauru*s *pacificus* (*sensu* [120]; for “*Shastasaurus altispinus*”: [239]). According to the analyses of Sander *et al.* ([235]) and Fröbisch *et al*. [236], *Callawayia* was not a shastasaurid, but instead more highly nested among the more advanced parvipelvian ichthyosaurs, the clade which is characterized by a different humerus shape, i.e., morphotype 3 *sensu* [237]. Motani [237] conceded, however, that a few taxa (e.g., *Cymbospondylus* and *Toretocnemus* Merriam, 1903 at the time) do not fit their proposed morphotype classification. Other recent phylogenetic analyses [199], [240], using updated versions of the data matrices of Motani [130] and Thorne *et al.* [193] and centering on the reinterpretation of grippidian material from the Sulphur Mountain Formation, British Columbia, as well as of *Utatsusaurus* from Japan, recovered *Callawayia* again in a more classical position in a clade with *Shastasaurus* and *Shonisaurus*.

If the systematic position of *Callawayia* as a parvipelvian ichthyosaur holds true [235], [236] and the new humerus is indeed assignable to this taxon, it would indicate that in addition to the more basal, non-ichthyosaurian ichthyopterygians, mixosaurids [241], and giant shastasaurids, also the more derived parvipelvian ichthyosaurs with thunniform ( =  tuna-like) body shapes were already present in the Early Triassic.

Triassic ichthyosaurs show a close correlation between humerus proximodistal length and overall body length of the animals (Fig. 3). Comparison of the new humerus (NMMNH P-65886) from Bear Lake County, Idaho, with the other Triassic ichthyosaurs such as *Cymbospondylus buchseri* Sander, 1989 from the Besano Formation, Monte San Giorgio, Switzerland [242], *C. piscosus* Leidy, 1868 from the Prida Formation, Nevada, USA [243], [244], *Shonisaurus popularis* (Kosch, 1990) from the Late Triassic Luning Formation of Nevada, North America [245], [246], and the shastasaurid *Guizhouichthyosaurus tangae* Cao & Luo in Yin *et al*., 2000 from the Late Triassic of China ([13], [247], the same specimen YIGMR TR00001 was figured as “*Panjiangsaurus epicharis*” in [248]; another specimen referred to as “*Shastasaurus*” *tangae* [IVPP V 11853]: [249]), indicated that NMMNH P-65886 did belong to an animal of about 11 meters in length (Fig. 3, Table S3 in File S1).

Another giant form, *Himalayasaurus tibetensis* Dong, 1972 from the Norian (Late Triassic) of Tibet, China, estimated to be about 15 meters in length [250], [251] could not be added to the analysis, because it lacks a humerus for comparison. Instead, if *Temnodontosaurus trigonodon* (Theodori, 1854), a giant species from the Lower Jurassic of Europe that may have exceeded nine meters or more in total body length [120], [252], [253] is considered, it would provide further support for the validity of our size estimation, since it falls close to the regression line of the documented Triassic data ([252] presented data on specimen SMNS 15950: 786 cm body length, ca. 21.5 cm humerus length [morphotype 3]; data not included in Fig. 3 because only Triassic taxa are shown).

With regard to very large ichthyosaurs such as *Shastasaurus liangae* (see Yin in [247]) from the Late Triassic of southwestern China and *S. sikanniensis* from British Columbia, suction feeding has been proposed as a possible mode of feeding similar to that of modern tooth-less whales, i.e., the baleen whales or ziphiid beaked whales [234], [235]. A recent comparative and biomechanical study of hyoid apparatuses and snout forms in Triassic and Early Jurassic ichthyosaurs, however, argues against this possibility, instead indicating that all proposed “suction-feeders” are actually typical “ram-feeders” [254]. It is not known, whether the new Spathian ichthyosaur humerus from Bear Lake County belonged to a true suction-feeding form or had a ram-feeding predatory lifestyle such as *Shonisaurus* or *Temnodontosaurus*. Independent of its ecology, the new specimen is important because it demonstrates that the Early Triassic ichthyosaur record is already quite diverse and that large ichthyosaurs, probably cruisers with higher metabolic rates that inhabited more open waters [255], [256], which are more derived than the non-ichthyosaurian ichthyopterygians known from that time, were already widespread during the Spathian.

#### Hupehsuchia

*Nanchangosaurus suni* Wang, 1959 and *Hupehsuchus nanchangensis* Young & Dong, 1972, the two taxa currently included in Hupehsuchia Carroll & Dong, 1991, were either recovered from the Jialingjiang Formation or from the upper part of the Daye limestone of Hubei Province, China [198], [257], [258]. The age of these fossils is estimated to range from late Early Triassic [16], [168], [256], [258], [259] to early Middle Triassic [198], [257], [260]). Wu *et al.* [260] for example noted an estimated age of “about 242 million years” for *Nanchangosaurus*, which would correspond to the late Anisian, but these authors referred to it as Early Triassic in age. Li *et al.* [168] on the other hand, argued for an Early Triassic (Olenekian) age, indicating that *Nanchangosaurus* may actually be slightly older than *Hupehsuchus*. Nevertheless, accurate age dating of these fossils remains difficult [169]. Furthermore, the reader should be aware that accurate age dating of these fossils is currently equivocal, depending on the definition of the Olenekian-Anisian boundary [36]. Similarly, very little is known about the dietary preferences of both taxa [257]. The systematic position of Hupehsuchia are presently not well understood, even though *Hupehsuchus* was recovered as sister taxon to Ichthyopterygia before [130].

## Discussion

Our results show that the prolonged step-wise recovery pattern of marine ecosystems following the end-Permian mass extinction as recently presented ([20]: Fig. 4) is incorrect and is in need of reconsideration as it does not reflect the global pattern. Outside China, there is ample evidence for large chondrichthyans and bony fishes as well as unusual marine temnospondyls shortly after the mass extinction, suggesting an early radiation of marine predators. Measurements of fishes show that the median body length significantly decreased from around 20 cm during the Early Triassic to about 15 cm in the Anisian (Mann-Whitney U test, p<0.01). The overall range of fish body sizes remained similar but the distribution of body length also significantly decreased (Kolmogorov-Smirnov test, p<0.01) between the Early Triassic and Anisian (Fig. 1). This reduction in body size is mainly the result of the diversification of small actinopterygian taxa (“subholosteans” and neopterygians) [52]. Fluctuations in fish body size distribution are observed at the sub-stage level during the Early Triassic, which may be due to lower sample size and differences in geographic sampling (Fig. 1). Marine tetrapods on the other hand show a non-significant (Mann-Whitney U and Kolmogorov-Smirnov tests) increase in body size between the Early Triassic and the Anisian (Fig. 1).

The fossil record clearly documents that already in Griesbachian and Dienerian times (Induan, earliest Triassic, Fig. 4A), global marine ecosystems did not consist of primary producers exclusively ([20]: p. 380, Fig. 3), but rather exhibited several trophic levels up to and including the presence of large aquatic vertebrates such as predatory bony fishes (e.g., *Saurichthys*, *Birgeria*, *Rebellatrix*, Fig. 4), chondrichthyans (e.g. *Hybodus*, Eugeneodontiformes, Fig. 4) and marine temnospondyl ‘amphibians’, such as the gharial-like lonchorhynchine trematosauroids with elongated slender snouts (e.g., *Aphaneramma*, *Wantzosaurus*, Fig, 4); faunal elements that would fall in the category P_2_ (“predatory fishes and reptiles”) of the trophic pyramid depicted in [20] (p. 379, Fig. 2). Although the marine temnospondyls are not specifically listed in the definition of category P_2_, these animals nevertheless still preyed upon smaller organisms of levels P1 (“predatory invertebratesr”) and P_2_, analogous to the larger predatory reptiles [20]. None of the Early Triassic species of marine trematosauroid temnospondyls are dominant over other taxa and they can therefore not represent “disaster taxa” (*sensu* [261]). The group ranged from the temperate zone in the North (Greenland and Spitsbergen, which were not in the polar region as they are today) to the temperate zone in the South (e.g., Madagascar) and must thus be considered supra-regionally to even globally distributed in the Early Triassic. Following Kauffmann and Harries ([261]: p. 21), the marine temnospondyls are instead interpreted herein as “crisis progenitors”, which “initially adapted to perturbed environmental conditions of the mass extinction interval, readily survive this interval, and are among the first groups to seed subsequent radiation into unoccupied ecospace during the survival and recovery intervals”. The same applies for fishes: Although there are many cosmopolitan genera during the Early Triassic [56], [59], [91], the taxonomic composition of the faunas is well-balanced, with no taxon predominating in terms of fossil abundance [102].

In order for a diversity of large marine predators to exist in the Early Triassic, at least a minimum interaction between primary producers, primary and secondary consumers (e.g., smaller fishes, conodonts, ammonoids) as well as the higher levels in the food web is essential, because these animals could not have thrived on the broader but lower trophic levels alone. This reasoning is supported by the body size survey of fishes in the Early Triassic and Anisian, which, if taken as proxies for trophic level [67], clearly support the existence of a multilevel trophic pyramid from the Griesbachian onwards. Thus, there is no basis for the claim ([20]: p. 379–380) that “[…] ecosystems were constructed step by step from low to top trophic levels through Early–Middle Triassic times […]”.

The recent publications addressing the recovery of marine trophic networks following the end-Permian event [11], [20] are further biased by the focus on conditions in China only rather than on a global perspective, and thus they do not fully represent the current state of research. This is for the most part due to the omission of fossil data and older literature, especially concerning groups not (yet) known in China, which has fundamental effects on the timing of recovery of the food chains after the end-Permian mass extinction. Equally important data from non-Chinese Early Triassic localities (e.g., Svalbard, Madagascar, Greenland, western Canada) that may contradict their presented delayed recovery patterns are less prominently discussed. Such data sets, especially pertaining to larger marine vertebrates [59] [121], [154], must be included into the discussion regarding biotic recovery patterns. Additional to theoretical modeling, research on coprolites (see below) and gut contents, Ca-isotope analyses and functional studies on feeding mechanics (e.g., geometric morphometric and finite element analyses, tooth wear analyses) would help to better understand the role of the different predatory guilds in Early Triassic food webs, but such data are currently not readily available in the literature. However, a delayed recovery of higher trophic levels within oceanic food webs following the end-Permian mass extinction can already be refuted based on the known fossil record of marine predatory vertebrates.

### Direct and Indirect Evidence for Predation

In addition to the direct evidence from the fossil record, indirect evidence is accumulating that suggests secondary marine reptiles likely evolved during the earlier stages of the Early Triassic. This is inferred from the high degree of adaptation to the aquatic environment, which is already present in the earliest known members of several of these independent marine reptilian lineages [121], [222]. If we consider the well-studied mammalian examples of sirenian and cetacean evolution during the early Cenozoic as analogues, a time span of 5 to 10 million years could be plausible for a transition from predominantly terrestrial animals to fully aquatic forms with streamlined body shapes and paddle-like limbs [262], [263]. Among Cetacea, the group including modern whales and dolphins, the transition from terrestrial ( =  land-living) *Pakicetus* Gingerich & Russell, 1981 (Early Eocene) to the first marine whales such as *Rodhocetus* Gingerich, Raza, Arif, Anwar & Zhou, 1994 or *Georgiacetus* Hulbert, Petkewich, Bishop, Bukry & Aleshire, 1998 (late Middle Eocene) required only about 5 million years [263]–[266]. Sirenia (sea cows), the only other extant group of fully aquatic mammals, began a similar transition from predominantly terrestrial to fully aquatic forms. *Prorastomus* Owen, 1855 (late Early and early Middle Eocene [267]) and *Pezosiren* Domning, 2001 (early Middle Eocene [268]) maintained semi-aquatic lifestyles. Fully aquatic forms such as *Protosiren* Abel, 1904 (Middle Eocene) retained hind limbs, even though they were probably no longer suited for terrestrial locomotion, whereas those of the extant dugongs and manatees eventually became completely reduced (as in whales, remnants of pelvic bones are still present; [262]).

If higher evolutionary rates are invoked for the Mesozoic marine reptiles, this would pointedly reduce the time span inferred for similar adaptations among the reptiles during the Early Triassic, however, one still has to expect these diverse lineages to be present shortly after the Permian-Triassic boundary, even though body fossils are not yet known from layers of earliest Triassic age. This is especially true for more derived early Spathian ichthyosaurs that, in addition to already having evolved large, streamlined bodies over 10 meters in total length (Fig. 3) and highly modified flipper morphologies only 2 myrs after the end-Permian extinction event [33], [269], would also have experienced profound physiological and developmental adaptations (indicated in Fig. 4B by tail of large ichthyosaur, no. 15, reaching into the left-hand side of image). One such change, namely the reproductive modification from oviparity to viviparity (or ovoviviparity), would certainly have to be obligatory in this lineage [259], [270]. It cannot be ruled out however that (ovo-)viviparity might have been already present in the terrestrial ancestors of the ichthyosaurs.

Another important source of information regarding predation is presented by phosphatic coprolites, some of which preserve fish remains (Fig. 2I-K; [271], [272]), Some Early Triassic specimens from marine deposits measure nearly 10 cm in length [135]. Obviously, such coprolites derive from large predatory aquatic vertebrates, and based on body fossils of similar stratigraphic age, the most likely producers are large fishes such as *Birgeria*, *Saurichthys* or coelacanths, temnospondyl ‘amphibians’ or marine reptiles.

### Sampling in the Early Triassic

According to the review-article of Chen & Benton [20], the authors do not consider the possibility that their “delayed recovery patterns” hypothesis following the end-Permian event could have been the result of sampling biases in Early Triassic sediments. With regard to larger marine vertebrates, we argue that biodiversity in the Early Triassic is underestimated for several reasons.

First of all, many marine reptilian lineages go through a shallow water phase before becoming fully pelagic [273]. Relatively smaller orbital and scleral ring diameters [259], as well as the apparent absence of bone collapse structures attributable to decompression syndrome corroborate the hypothesis that Triassic ichthyosaurs mainly inhabited shallow waters [274]. This is corroborated by the results of Cuthbertson *et al.* ([199]: p. 846) who, while discussing the yet elusive center of origin and early radiation of ichthyopterygians and presenting several possible pre-Olenekian dispersal routes, currently favour migration and dispersal “through shallow water regions between breaches in the otherwise continuous and contiguous continental landmasses”. Sections consisting of pelagic sediments, on the other hand, are usually studied more thoroughly because they often contain biostratigraphically important invertebrate fossils such as ammonoids, but they may not necessarily be the most likely sediments for preservation of these early forms of marine reptiles. Furthermore, the fossilization potential of marine vertebrates in sediments derived from near-shore environments will likely be reduced due to mechanical separation and disarticulation by wave action or scavenging.

Secondly, numerous sections that are known to contain Early Triassic marine vertebrates (or may potentially yield them) are restricted to remote areas (e.g., in polar regions such as Greenland and Spitsbergen) and thus are not as heavily sampled as other, more easily accessible areas. On the other hand, many classical, easily accessible, European Middle Triassic localities for marine vertebrates have been extensively researched for more than 150 years (e.g. Monte San Giorgio in southern Switzerland, German Muschelkalk Sea). Furthermore, many Early Triassic vertebrate fossils such as bony fishes are recovered from early diagenetic limestone nodules, which are restricted in size. Because of a lack of diagnostic characters, larger, incomplete specimens can often be determined only to higher taxonomic levels, thus distorting the actual diversity patterns.

Thirdly, locations yielding Early Triassic vertebrate fossils are still being recovered. Although the presence of Triassic marine reptiles from China has been known since the 1950s [275], it has only been during the last two decades that the global importance of the various reptile-bearing Triassic black shales of southern and southwestern China has been recognized [11], [13], [16], [276]. Even though marine Triassic fishes from China have been described sporadically during the last century [277]–[279], they have experienced increased attention by researchers over the last decade [60], [63], [64], [103], [104], [280]–[282]. Indeed, the fact that the Middle Triassic Luoping biota [18] was only recently discovered, well demonstrates that such ‘sampling biases’ in the fossil record can persist for a long time. Since its discovery, the extensive large-scale quarrying of the fossiliferous layers at the Luoping site has led to an amazing amount of fossil specimens (nearly 20000 recovered macrofossils in 2011 but most are yet to be described [11], [18]), whereas sampling in other difficult to reach but potentially rewarding places such as Spitsbergen or Greenland is based mainly on sporadic, very expensive expeditions, where conditions generally allow for surface collecting only.

Fourthly, possibilities remain that Permian taxa might be recovered from Early Triassic sediments as well. For instance, in the 1920s, a diverse reptile fauna was reported from the Upper Permian Lower Sakamena Formation of Madagscar [283]–[285] that included procolophonoid and tangasaurid “younginiform” diapsids. Recently, however, new fossils typical of these predominantly Paleozoic reptiles were described from Early Triassic sediments (Middle Sakamena Formation), either from the “Couches à *Claraia* et Poissons” or “Couches à Poissons et Ammonites” horizon, Diego Basin, northwestern Madagascar [286], which correspond to the local Otoceratan (≈ Griesbachian) or Gyronitian (≈ Dienerian) age, respectively [287]. If this age assignment holds true, the presence of the near-shore marine *Hovasaurus boulei* Piveteau, 1926 ([284], [285], see Fig. 4) as a potential survivor of the end-Permian mass extinction event is important for the present discussion. As a small-bodied reptile well-adapted to the aquatic environment (e.g., stomach stones as bone ballast; well-developed elongated swimming tail; pachyostotic ribs; [283], [284]), it can be viewed as an Early Triassic analogue in terms of anatomy and ecology to the abundant pachypleurosaurid sauropterygians, which diversified later during the Triassic. These new discoveries therefore would yet increase the diversity of taxa present in the earliest Early Triassic food web. Another example of this kind would be the recent discovery of the typically Paleozoic chondrichthyan *Listracanthus* in the Early Triassic of Canada [84].

Finally, due to their position in the trophic web, apex predators are usually much rarer than primary and secondary consumers in the fossil record. If the remains of apex predators are, however, recovered in relatively large numbers, as is for example the case for ichthyosaurs and trematosauroids from the Early Triassic of Spitsbergen, Greenland and Madagascar [120], [135], [144], [152], [191], this argues against a truncation of the higher trophic levels.

### The Predators’ Influence on Ecosystems

Where present in the Early Triassic, a disturbed “evenness” with dominance of individual species over others (see above) is not recognized among marine fishes or the larger marine tetrapod lineages. Instead it appears that many of the novel “numerous predatory levels” proposed for the Luoping biota [20] were already present during the Early Triassic (Table 1). This categorization scheme is not interpreted as being exclusive, but instead, is indicative of trophic feeding preferences. It thus becomes apparent that even in the early stages of the Early Triassic, longer food chains than those previously proposed [20] must have been present, which argues against a delayed recovery of the upper levels within marine food webs after the end-Permian mass extinction.

**Table 1:** Predator-prey relationships during the Early Triassic.

	durophagous predators	Small and mid-sized carnivores	larger carnivores
**Prey items:**	Invertebrates (e.g., cephalopods, gastropods, bivalves, crustaceans)	Invertebrates	Invertebrates
		conodonts?	conodonts?
		fishes	fishes
			smaller carnivores (e.g., juvenile trematosauroids and reptiles)
**Vertebrate predators:**	**chondrichthyan fishes**	**chondrichthyan fishes**	**chondrichthyan fishes**
	*Acrodus*, *Palaeobates*	*Hybodus*	*Hybodus*, Eugeneodontiformes
	**actinopterygian fishes**	**actinopterygian fishes**	**actinopterygian fishes**
	*Bobasatrania*	*Birgeria*, *Saurichthys*	*Birgeria, Saurichthys*
	**actinistian fishes**	**actinistian fishes**	**ichthyosaurs**
	*Mylacanthus*, *Scleracanthus*	*Rebellatrix*	*Pessopteryx*, *Quasianosteosaurus*, *?Callawayia*-like ichthyosaurs
	**ichthyosaurs**	**thalattosaurs**	**temnospondyl ‘amphibians’**
	*Omphalosaurus, Chaohusaurus*	*Paralonectes*, *Agkistrognathus*	*Svalbardosaurus*
		**trematosauroid ‘amphibians’**	
		*Aphaneramma*, *Wantzosaurus*	
		**sauropterygians**	
		*Corosaurus*, *?Kwangsisaurus*	
		**ichthyosaurs**	
		*Utatsusaurus, Grippia*	
		***hupehsuchians*** **?**	
		*Hupehsuchus, Nanchangosaurus*	

Only a few examples are given for each group. Note that even though conodonts are not listed specifically, they nevertheless would have contributed to the ancient food webs as both predators and prey. See text for references.

The omission of the large marine temnospondyls, and the neglection of the global Early Triassic record of large predatory fishes in the discussion concerning the reconstruction of marine food chains and the associated timing of the recovery following the end-Permian event erroneously led to the conclusion [20] that higher trophic levels were absent until Middle Triassic times. The widespread presence of large marine predators indicates that numerous prey such as small fishes, conodonts, crustaceans and molluscs (primary and secondary consumers) must have been abundant in the Early Triassic, suggesting that multi-level trophic networks were already established shortly after the end-Permian event, although the taxonomic composition was different from that prior to the mass extinction. Furthermore, the larvae and juveniles of predatory fishes probably fed on different prey items than adult individuals (e.g. eggs and larvae of other animals, ostracods), thus presumably adding more complexity to the trophic network. If juvenile trematosauroids were not exclusively piscivorous, but instead, were opportunistic feeders preying upon a variety of smaller animals including invertebrates (Table 1), this would add yet another level of predator-prey interaction to the system. It is noteworthy that despite their abundance elsewhere in the Early Triassic, trematosauroid temnospondyls have yet to be discovered in sediments of that age in China [122], [154].

Early Triassic ecosystems excluding those of China contain an array of predatory organisms (see above, Fig. 4). The conclusion that marine ecosystems immediately following the PT-mass extinction “[…] were degraded to a low level, typified by primary producers or opportunistic consumers […]” ([20]; p. 379) lacks global support, and could perhaps be a peculiarity of the Chinese fossil record, in the worst of all cases. As is clearly evident in the fossil record, the marine trophic pyramid was not truncated in the Early Triassic. The presence of multiple trophic levels at the onset of the Triassic (Griesbachian) is supported by the variety of shapes and body sizes of marine predators, as well as their global distribution and abundance in the fossil record. After the loss of the dominant Paleozoic marine apex predators (mainly chondrichthyans, e.g., [20], [24], [26], [52]) during the PT mass extinction, the higher levels of the trophic pyramid were rapidly occupied by other vertebrate groups, first mainly by temnospondyl ‘amphibians’ and fishes and later predominantly by marine reptiles and fishes (Fig. 4). The appearance of secondary marine reptiles as apex predators in the Triassic cannot be used as a metric for the timing of biotic recovery [20], since, as stated by the authors, these groups were not among the dominant apex predators in the ocean before the mass extinction event. Secondary marine reptiles instead should be regarded as an evolutionary novelty, just as the marine trematosauroid ‘amphibians’ that appeared earlier. Although taxonomic richness of marine vertebrates continued to rise from the Early to the Middle Triassic (e.g., [26]–[28], [117], [124]), and hence the complexity of trophic networks probably increased likewise, we emphasize that the diversity among Early Triassic apex predators as well as their prey already allowed for a variety of trophic interactions and food chains of usual lengths.

### The Smithian-Spathian Turnover of Apex Predators

The emergence of some of the dominant marine reptiles of the Mesozoic, i.e. ichthyopterygians and sauropterygians, as apex predators in the Spathian roughly coincides with the disappearance of marine temnospondyl ‘amphibians’ and eugeneodontiform sharks. Marine temnospondyls are predominantly known from the Griesbachian, Dienerian and Smithian sub-stages of the Early Triassic [122], [154], [288], where they had a global distribution (Fig. 4, Table S2 in File S1), although there is indication that an Anisian trematosauroid from Jordan might have inhabited also shallow marine coastal regions [289]. Early Triassic eugeneodontiform remains, where well-dated, are usually from horizons older than the Spathian [78], [79], [82]. This turnover among marine apex predators falls within the Smithian-Spathian transition (Fig. 4), which is well-known for the major near-extinction of nekto-pelagic clades such as ammonoids and conodonts (e.g., [50], [290], [291]). The Smithian-Spathian boundary crisis was linked with a profound climatic change from warmer and more humid conditions during the Smithian to cooler and dryer conditions in the Spathian ([4] and references therein). The processes by which the Smithian-Spathian-boundary event [292], approximately 2 myr after the main extinction pulse near the Permian-Triassic boundary, modulated the Early Triassic apex predator turnover remains to be explored.

A similar turnover scenario may have been linked to the end-Permian mass extinction when the dominant marine predators of the Late Paleozoic (mainly fishes) were replaced by trematosauroid and other temnospondyl ‘amphibians’, as well as by new taxa of predatory osteichthyans (*Birgeria*, *Saurichthys*, *Rebellatrix*). If we examine the continental vertebrate record in the world-famous Karoo section in southern Africa for comparison, we note that the end-Permian extinction event appeared to be selective in that certain tetrapod lineages suffered more than others; whereas the decrease in overall generic diversity of terrestrial vertebrates is related mainly to the severe decline in synapsid diversity and also to a small degree to the decline of fish generic richness, the diversity of ‘amphibians’ and reptiles actually increases across the Permian-Triassic boundary ([26], [5]: Fig. 1A). This increase in diversity of both groups may have supported the colonization of near-shore environments during the Early Triassic, first by the temnospondyl ‘amphibians’ and some two millions years later by secondary marine reptiles.

## Conclusions

1. Global fossil evidence clearly demonstrates that marine apex predators were always present during the earliest Triassic (from the Griesbachian onward), thus emphasizing the regeneration and/or inheritance of full length, multi-level trophic food webs immediately after the end-Permian mass extinction.
2. Spatial and stratigraphic distribution of marine predatory vertebrates (fishes, temnospondyl ‘amphibians’, and reptiles) does not support a step-wise recovery model of Triassic trophic webs.
3. A sharp faunal turnover among marine predatory guilds during the Early Triassic is apparent and was centered around the Smithian-Spathian boundary, because those ecosystems with predominantly trematosauroid temnospondyl ‘amphibians’ and fishes as apex predators switched to ecosystems with marine reptiles (ichthyosaurs, sauropterygians, thalattosaurs, protorosaurians) and fishes at the uppermost end of the food chain.
4. The disturbance of ecosystems during and after the Permian-Triassic mass extinction event may have triggered the evolution and early diversification of marine vertebrate groups such as actinopterygian fishes, as well as secondary marine temnospondyl ‘amphibians’.
5. There is no significant increase in body size of marine apex predators (fishes, tetrapods) from the Early Triassic to the Anisian (early Middle Triassic), invalidating previous assumptions of a step-wise recovery of the trophic pyramid after the end-Permian event.

## Supporting Information

File S1 Table S1. Xcel-spreadsheet with maximum standard lengths of marine species of bony fishes (Actinistia, Actinopterygii) in the Early Triassic and the Anisian (Middle Triassic) based on literature data. Table S2. Xcel-spreadsheet with list of tetrapod species surveyed and accompanying occurrence and maximum size ( =  total length) data. The compilation of Middle Triassic taxa is based on Kelley *et al*. (2012) [124] with some taxa, reference and size data modified or added. Table S3. Xcel-spreadsheet with data used for the humeral proximodistal length-body length relation in Triassic ichthyosaurs (Fig. 3).
(XLSX)

File S2 **Additional references accompanying Tables S1–S2 in File S1.**
(DOC)
