# Supplementary material for: Early Triassic Marine Biotic Recovery: The Predators' Perspective
Source: PLoS One. 2014 Mar 19;9(3):e88987. doi: 10.1371/journal.pone.0088987 (PMC3960099; doi:10.1371/journal.pone.0088987)
Supplement: File S2 — Additional references accompanying Tables S1–S2 in File S1. (DOC) [file pone.0088987.s002.doc]

**Appendix S2:** References accompanying Appendices S1-S2.

**References to Appendix S1:**

Abel O (1906) Fossile Flugfische. Jahrbuch der Kaiserlich-Königlichen Geologischen Reichsanstalt 56 (1): 1–88.

Alessandri Gd (1910) Studii sui pesci Triasici della Lombardia. Memorie della Società italiana di Scienza naturale 7(1): 1–147.

Andersson (= Stensiö) E (1916) Beschreibung einiger Fischreste aus Madagaskar und Siam. Bulletin of the Geological Institution of the University of Upsala 13: 227–232.

Bashkuev A, Sell J, Aristov D, Ponomarenko A, Sinitshenkova N. Mahler H (2012) Insects from the Buntsandstein of Lower Franconia and Thuringia. Paläontologische Zeitschrift 86(2): 175–185.

Bassani F (1886) Sui fossili e sull’età degli schisti bituminosi Triasici di Besano in Lombardia. Communicazione preliminare. Atti della Società Italiana di Scienze Naturale 29: 15–72.

Bellotti C (1857) Descrizione di alcune nuove specie di pesci fossili di Perledo e di altre località lombarde. In: Stoppani A, editor. Studii Geologici e Paleontologici sulla Lombardia. Milano: Turati. pp. 419–438.

Bellotti C (1873) Osservazioni alla collezione dei pesci fossili del Museo civico. Dal catalogo manoscritto del 1873: 27–30. [Reprinted in: Pinna, G (1991) I primi studi sui pesci fossili triassici della Lombardia al Museo di Storia Naturale di Milano. Natura: Rivista di Scienze Naturali 82: 1–41.]

Beltan L (1968) La faune ichthyologique de l’Eotrias du N.W. de Madagascar: le neurocrâne. CNRS, Paris: Cahiers de paléontologie. 135 p.

Beltan L (1977) La parturition d’un actinoptérygian de l’Eotrias du nord-ouest de Madagascar. Comptes Rendus Hebdomadaires des Séances de l’Académie des Sciences, Paris 284: 2223–2225.

Beltan L (1980) Eotrias du nord-ouest de Madagascar: Etude de quelques poissons, dont un est en parturition. Annales de la Société Géologique du Nord 99: 453–464.

Beltan L (1984) A propos d’un poisson Volant biplane de l’Eotrias du NW de Madagascar. Annales de la Société Géologique du Nord 103: 75–82.

Beltan L, Janvier P (1978) Un nouveau Saurichthyidae (Pisces, Actinopterygii), *Saurichthys nepalensis* n. sp., du Trias inférieur des Annapurnas (Thakkhola, Nepal) et sa signification paléobiogéographique. Cybium 3(4): 17–28.

Berg LS, Kazantseva AA, Obruchev DV (1967) Superorder Palaeonisci (Archistia). In: Obruchev DV, editor. Fundamentals of Paleontology, 11, Agnatha, Pisces. Jerusalem: Israel Program for Scientific Translation. pp. 528–573. [Translated from Russian, published in Russian 1964: Osnovy paleontologii]

Brough J (1939) The Triassic fishes of Besano, Lombardy. London: British Museum (Natural History). 117 p.

Bürgin T (1992) Basal ray-finned fishes (Osteichthyes; Actinopterygii) from the Middle Triassic of Monte San Giorgio (Canton Tessin, Switzerland). Systematic palaeontology with notes on functional morphology and palaeoecology. Schweizerische Paläontologische Abhandlungen 114: 1–164.

Bürgin T (1995) Actinopterygian fishes (Osteichthyes; Actinopterygii) from the Kalkschieferzone (Uppermost Ladinian) near Meride (Canton Ticino, southern Switzerland). Eclogae Geologicae Helvetiae 88(3): 803–826.

Bürgin T (2004) †*Eosemionotus ceresiensis* sp. nov., a new semionotiform fish (Actinopterygii, Halecostomi) from the Middle Triassic Monte San Giorgio (southern Switzerland). In: Arratia G, Tintori A, editors. Mesozoic Fishes 3, Systematics, Paleoenvironments and Biodiversity. Dr. Friedrich Pfeil, München. pp. 239–251.

Chang M-M, Miao D (2004) An overview of Mesozoic fishes in Asia. In: Arratia, G. & Tintori, A. (eds) Mesozoic Fishes 3. Systematics, Palaeoenvironments and Biodiversity. Dr. Friedrich Pfeil, München. pp. 535–563.

Clément G (1999) The actinistian (Sarcopterygii) *Piveteauia madagascariensis* Lehman from the Lower Triassic of northwestern Madagascar: A redescription on the basis of new material. Journal of Vertebrate Paleontology 19(2): 234–242.

Compter G (1891) Ein Beitrag zur Paläontologie des oberen Muschelkalkes. Zeitschrift für Naturwissenschaften 64: 41–61.

Corroy G (1928) Les vertébrés du Trias de Lorraine et le Trias Lorrain. Annales de Paléontologie 17: 83–136.

Dames W (1888) Die Ganoiden des deutschen Muschelkalks. Paläontologische Abhandlungen 4(2): 133–180.

Deecke W (1889) Ueber Fische aus verschiedenen Horizonten der Trias. Palaeontographica 35: 97–138.

Eck H (1865) Ueber die Formationen des bunten Sandsteins und des Muschelkalks in Oberschlesien und ihre Versteinerungen. Berlin: J.F. Stracke. 148 p.

Firtion F (1934) Description d’une nouvelle espèce de *Saurichthys* du Grès à Voltzia de Wasselonne. Bulletin du Service de la Carte géologique d’Alsace et de Lorraine 2: 89–97.

Forey PL (1998) History of the coelacanth fishes. London: Chapman & Hall. 419 p.

Frech F (1903–1908) Lethaea geognostica 2, Das Mesozoicum, 1. Trias. Stuttgart: Schweitzerbart’sche Verlagsbuchhandlung. 623 p.

Frickhinger KA (1991) Fossilien Atlas Fische. Melle: Mergus. 1088 p.

Furrer, H. (2009) The diet of the predator fish *Saurichthys* from two Middle Triassic Plattenkalk lagerstätten in the Swiss Alps. 5th International Symposium on Lithographic Limestone and Plattenkalk, Basel, Abstracts and Field Guides, 30–31.

Gall J-C, Grauvogel L, Lehman J-P (1974) Faune du Buntsandstein, V. Les poissons fossiles de la collection Grauvogel-Gall. Annales de Paléontologie 60(2): 129–147.

Gall J-C, Grauvogel-Stamm L (2005) The early Middle Triassic ‘Grès àVoltzia’ Formation of eastern France: a model of environmental refugium. Comptes Rendus Palevol 4: 637–652.

Gardiner BG (1960) A revision of certain actinopterygian and coelacanth fishes, chiefly from the Lower Lias. Bulletin of the British Museum (Natural History), Geology 4(7): 239–384.

Gardiner BG (1988) A new *Cleithrolepis* from the Triassic of central Cyrenaica, northeast Libya. In: El-Arnauti A, Owens B, Thusu B, editors. Subsurface Palynostratigraphy of Northeast Libya. Garyounis University Publications, Benghazi. pp. 259–265.

Griffith J (1959) On the anatomy of two saurichthyid fishes, *Saurichthys striolatus* (Bronn) and *S. curioni* (Bellotti). Proceedings of the Zoological Society of London 132: 587–606 [doi: 510.1111/j.1469-7998.1959.tb05538.x].

Guffroy S (1956) Notes paléoichthyologiques. Bulletins de la Société Géologique de France 6: 847–854.

Herzog A (2001) *Peltoperleidus obristi* sp. nov., ein neuer, kleiner Strahlenflosser (Actinopterygii, Perleidiformes) aus der Prosanto-Formation (Mitteltrias) von Graubünden (Schweiz). Eclogae Geologicae Helvetiae 94: 495–507.

Herzog A, Bürgin T (2005) A new species of the genus *Besania* Brough 1939 from the Middle Triassic of Canton Grisons (Switzerland) with a discussion of the phylogenetic status of the taxon. Eclogae Geologicae Helvetiae 98: 113–122.

Hilzheimer M (1930) Ein *Saurichthys* Ag. aus dem Röt von Rüdersdorf. Brandenburgia. Monatsblatt der Gesellschaft für Heimatkunde und Heimatschutz in der Mark Brandenburg 39: 18–20.

Hitij T, Tintori A, Žalohar J, Renesto S, Celarc B, Križnar M, Kolar-Jurkovšek T (2010) New fossil sites with Triassic vertebrate fauna from the Kamnik-Savinja Alps, Slovenia. International Symposium on Triassic and Later Marine Vertebrate Faunas, Beijing. Program and Abstract: 42–46.

Hutchinson P (1973) A revision of the redfieldiiform and perleidiform fishes from the Triassic of Bekker’s Kraal (South Africa) and Brookvale (New South Wales). Bulletin of the British Museum (Natural History), Geology 22(3): 236–354.

Jin F (2006) An overview of Triassic fishes from China. Vertebrata PalAsiatica 44: 28–42.

Jin F, Wang N-Z, Cai Z-Q (2003) A revision of the perleidid fishes from the Lower Yangtze region of south China. Vertebrata PalAsiatica 41(3): 169–184. [in Chinese with English summary]

Jörg E (1969) Eine Fischfauna aus dem Oberen Buntsandstein (Unter-Trias) von Karlsruhe-Durlach (Nordbaden). Beiträge zur Naturkundlichen Forschung in Südwestdeutschland 28: 87–102.

Kner R (1866) Die Fische der bituminösen Schiefer von Raibl in Kärnthen. Sitzungsberichte der Kaiserlichen Akademie der Wissenschaften, Mathematisch-naturwissenschaftliche Klasse 53: 152–197.

Kogan I (2011) Remains of *Saurichthys* (Pisces, Actinopterygii) from the Early Triassic Wordie Creek Formation of East Greenland. Bulletin of the Geological Society of Denmark 59: 93–100.

Kunisch H (1885) *Dactylolepis gogolinensis* nov. gen., nov. spec.: Zeitschrift der Deutschen Geologischen Gesellschaft 37: 588–594.

Lambe LM (1914) Description of a new species of *Platysomus* from the neighborhood of Banff, Alberta. Transactions of the Royal Society of Canada, Series 3, Section 4, 8: 17–23.

Lambe LM (1916) Ganoid fishes from near Banff, Alberta. Proceedings and Transactions of the Royal Society of Canada, Series 3, 10(4): 35–44.

Lehman J-P (1952) Etude complémentaire des poissons de l’Eotrias de Madagascar. Kungliga Svenska Vetenskapsakademiens Handlingar 4: 1–192.

Lehman J-P (1953) Etude d’un *Perleidus* du Trias de Madagascar. Annales de Paléontologie 39: 3–18.

Lehman J-P (1956) Compléments à l’étude des genres *Ecrinesomus* et *Bobasatrania* de l’Eotrias de Madagascar. Annales de Paléontologie 42: 67–94.

Lehman J-P, Château C, Laurain M, Nauche M (1959) Paléontologie de Madagascar, 27, Les poissons de la Sakamena moyenne. Annales de Paléontologie 45: 175–219.

Li Q (2009) A new parasemionotid-like fish from the Lower Triassic of Jurong, Jiangsu Province, South China. Palaeontology 52: 369–384.

Lin H, Sun Z, Tintori A, Lombardo C, Jiang D, Hao W (2011) A new species of *Habroichthys* Brough, 1939 (Actinopterygii; Peltopleuriformes) from the Pelsonian (Anisian, Middle Triassic) of Yunnan Province, South China. Neues Jahrbuch für Geologie und Paläontologie, Abhandlungen 262: 79–89.

Liu G-B, Feng H-Z, Wang J-X, Wu T-M, Zhai Z-H (2002) Early Triassic fishes from Jurong, Jiangsu [in Chinese with English summary]. Acta Palaeontologica Sinica 41: 27–52.

Lombardo C (1995) *Perleidus altolepis* (Actinopterygii, Perleidiformes) from the Kalkschieferzone of Ca’ del Frate (N. Italy). Géobios 19: 211–213.

Lombardo C (2001) Actinopterygians from the Middle Triassic of northern Italy and Canton Ticino (Switzerland): Anatomical descriptions and nomenclatural problems. Rivista Italiana di Paleontologia e Stratigrafia 107(3): 345–369.

Lombardo C, Rusconi M, Tintori A (2008) New perleidiform from the Ladinian (Middle Triassic) of the northern Grigna (northern Italy). Rivista Italiana di Paleontologia e Stratigrafia 114(2): 263–272.

Lombardo C, Sun ZY, Tintori A, Jiang DY, Hao WC (2011) A new species of *Perleidus* (Actinopterygii) from the Middle Triassic of Southern China. Bollettino della Società Paleontologica Italiana 50(2): 75–83.

López-Arbarello A, Sun Z-Y, Sferco E, Tintori A, Xu G-H, Sun Y-L, Wu F-X, Jiang D-Y (2011). New species of *Sangiorgioichthys* Tintori and Lombardo, 2007 Neopterygii, Semionotiformes) from the Anisian of Luoping (Yunnan Province, South China). Zootaxa 2749: 25–39.

Minikh AV (1981) *Saurichthys* from the Triassic of the USSR [Zaurikhtisy iz triasa SSSR]. Paleontologicheskiy Zhurnal 1981(1): 105–113. [in Russian]

Minikh AV (1982) New species of *Saurichthys* from the Early Triassic from the area along the upper Volga [Novye vidy zaurichtisov iz rannego triasa verkhnego Povolzhya]. Ezhegodnik Vsesoyuzhnogo Paleontologicheskogo Obshchestva 25: 205–213. [in Russian]

Moy-Thomas JA (1935) The coelacanth fishes from Madagascar. Geological Magazine 72(5): 213–227.

Müller AH (1969) Über *Dollopterus volitans* (Osteichthyes, Chondrostei), einen Flugfisch aus dem germanischen Oberen Muschelkalk sowie einige Bemerkungen zur Biostratinomie und zur Konkretionsbildung. Freiberger Forschungshefte C 256: 37–46.

Mutter RJ (2001) The skull of Colobodontidae sensu Andersson 1916 (emended) (Actinopterygii: Perleidiformes). Geologia Insubrica 6(1): 65–78.

Mutter RJ (2002) Revision of the Triassic family Colobodontidae sensu Andersson 1916 (emended) with a tentative assessment of perleidiform interrelationships (Actinopterygii: Perleidiformes). PhD thesis, University of Zurich, Switzerland. 335 pp.

Mutter RJ (2004) The “perleidiform” family Colobodontidae: A review. In: Arratia G, Tintori A, editors. Mesozoic Fishes 3, Systematics, Paleoenvironments and Biodiversity. München: Dr. Friedrich Pfeil. pp. 197–208.

Mutter RJ (2005) Re-assessment of the genus *Helmolepis* Stensiö 1932 (Actinopterygii: Platysiagidae) and the evolution of platysiagids in the Early-Middle Triassic. Eclogae Geologicae Helvetiae 98: 271–280.

Mutter RJ, Cartanyà J, Basaraba SAU (2008) New evidence of *Saurichthys* from the Lower Triassic with an evaluation of early saurichthyid diversity. In: Arratia G, Schultze H-P, Wilson MVH, editors. Mesozoic Fishes 4 Homology and Phylogeny. München: Dr. Friedrich Pfeil. pp. 103–127.

Mutter RJ, Herzog A (2004) A new genus of Triassic actinopterygian with an evaluation of deepened flank scales in fusiform fossil fishes. Journal of Vertebrate Paleontology 24(4): 794–801.

Mutter RJ, Neuman AG (2008) New eugeneodontid sharks from the Lower Triassic Sulphur Mountain Formation of Western Canada. In: Cavin L, Longbottom A, Richter M, editors. Fishes and the Break-up of Pangaea Geological Society, London, Special Publications, 295. pp. 9–41.

Mutter RJ, de Blanger K, Neuman AG (2007) Elasmobranchs from the Lower Triassic Sulphur Mountain Formation near Wapiti Lake (BC, Canada). Zoological Journal of the Linnean Society 149: 309–337.

Mutter RJ, Neuman AG, de Blanger K (2008) *Homalodontus* nom. nov., a replacement name for *Wapitiodus* Mutter, de Blanger and Neuman, 2007 (Homalodontidae nom. nov., ?Hybodontoidea), preoccupied by *Wapitiodus* Orchard, 2005. Zoological Journal of the Linnean Society 154: 419–420.

Neuman AG (1986) Fossil fishes of the families Perleididae and Parasemionotidae from the Lower Triassic Sulphur Mountain Formation of Western Canada. MSc Thesis, Edmonton: University of Alberta. 142 p.

Neuman AG, Mutter RJ (2005) *Helmolepis cyphognathus*, sp. nov., a new platysiagid actinopterygian from the Lower Triassic Sulphur Mountain Formation (British Columbia, Canada). Canadian Journal of Earth Sciences 42: 25–36.

Nielsen E (1936) Some few preliminary remarks on Triassic Fishes from East Greenland. Meddelelser om Grønland 112(3): 1–55.

Nielsen E (1942) Studies on Triassic fishes from East Greenland 1. *Glaucolepis* and *Boreosomus*. Palaeozoologica Groenlandica 1: 1–403.

Nielsen E (1949) Studies on Triassic fishes from East Greenland 2. *Australosomus* and *Birgeria*. Palaeozoologica Groenlandica 3: 1–309.

Nielsen E (1952) A preliminary note on *Bobasatrania groenlandica*. Meddelelser fra Dansk Geologisk Förening 12(12): 197–204.

Nybelin O (1977) Studies on Triassic fishes from East Greenland III. On *Helmolepis gracilis* Stensiö. Meddelelser om Grønland 200(2): 1–13.

Oertle GF (1927) „*Semionotus letticus* O. Fraas“ und andere Ganoiden aus dem Hohenecker Kalk, von innen gesehen. Neues Jahrbuch für Mineralogie, Geologie und Paläontologie, Abteilung B 58: 309–334.

Oertle GF (1928) Das Vorkommen von Fischen in der Trias Württembergs. Neues Jahrbuch für Mineralogie, Geologie und Paläontologie, Abteilung B 60: 325–472.

Oosterink HW (1986) Winterswijk, geologie 2. De trias-periode (geologie, mineralen en fossielen). Wetenschappelijke Mededelingen van de Koninklijke Nederlandse Natuurhistorische Vereniging 178: 1–120.

Piveteau J (1934) Paléontologie de Madagascar XXI. – Les poissons du Trias inférieur. Contribution à l’étude des actinoptérygiens. Annales de Paléontologie 23: 83–178.

Piveteau J (1939–1940) Paléontologie de Madagascar XXIV. – Nouvelles recherches sur les poissons du Trias inférieur. Annales de Paléontologie 28: 71–88.

Piveteau J (1944-1945) Paléontologie de Madagascar XXV. - Les poissons du Trias inférieur, la famille des Saurichthyidés. Annales de Paléontologie 31: 79–89.

Priem F (1924) Paléontologie de Madagascar XII. Les poisons fossiles. Annales de Paléontologie 13: 107–132.

Qian MP, Zhu SP, Zhao FM, Zhou XD, Su R, Gu GH, Zhai ZH (1997) Discovery of Early Triassic fish fossils and its significances in Jurong, Jiangsu Province. Jiangsu Geology 21(2): 65–71. [In Chinese with English abstract]

Raymond PE (1925) Two new fossil fishes from Alberta. The American Journal of Science 5(9): 551–555.

Reis OM (1892) Zur Osteologie und Systematik der Belonorhynchiden und Tetragonolepiden. Geognostische Jahreshefte 4: 143–171.

Rieppel O (1980a) Additional specimens of *Saurichthys madagascariensis* Piveteau, from the Eotrias of Madagascar. Neues Jahrbuch für Geologie und Paläontologie, Monatshefte 1980(1): 43–51.

Rieppel O (1980b) A new coelacanth from the Middle Triassic of Monte San Giorgio, Switzerland. Eclogae Geologiae Helvetiae 73(3): 921–939.

Rieppel O (1982) A new genus of shark from the Middle Triassic of Monte San Giorgio, Switzerland. Palaeontology 25(2): 399–412.

Rieppel O (1985) Die Triasfauna der Tessiner Kalkalpen 25. Die Gattung *Saurichthys* (Pisces, Actinopterygii) aus der mittleren Trias des Monte San Giorgio, Kanton Tessin. Schweizerische Paläontologische Abhandlungen 108: 1–103.

Rieppel O (1992) A new species of the genus *Saurichthys* (Pisces: Actinopterygii) from the Middle Triassic of Monte San Giorgio (Switzerland), with comments on the phylogenetic interrelationships of the genus. Palaeontographica Abt A 221: 63–94.

Romano C, Brinkmann W (2009) Reappraisal of the lower actinopterygian *Birgeria stensioei* ALDINGER, 1931 (Osteichthyes; Birgeriidae) from the Middle Triassic of Monte San Giorgio (Switzerland) and Besano (Italy). Neues Jahrbuch für Geologie und Paläontologie, Abhandlungen 252: 17–31 [doi: 10.1127/0077-7749/2009/0252-0017].

Romano C, Brinkmann W (2010) A new specimen of the hybodont shark *Palaeobates polaris* with three-dimensionally preserved Meckel's cartilage from the Smithian (Early Triassic) of Spitsbergen. Journal of Vertebrate Paleontology 30: 1673–1683.

Romano C, Kogan I, Jenks J, Jerjen I, Brinkmann W (2012) *Saurichthys* and other fossil fishes from the late Smithian (Early Triassic) of Bear Lake County (Idaho, USA), with a discussion of saurichthyid palaeogeography and evolution. Bulletin of Geosciences 87: 543–570 [doi: 510.3140/bull.geosci.1337].

Russell LS (1951) *Bobasatrania*? *canadensis* (Lambe), a giant chondrostean fish from the Rocky Mountains. National Museum of Canada Bulletin 123: 218–224.

Sander PM, Rieppel OC, Bucher H (1994) New marine vertebrate fauna from the Middle Triassic of Nevada. Journal of Paleontology 68(3): 676–680.

Schaeffer B, Mangus M (1976) An Early Triassic fish assemblage from British Columbia. Bulletin of the American Museum of Natural History 156: 515–564.

Schultze H-P, Möller H (1986) Wirbeltierreste aus dem Mittleren Muschelkalk (Trias) von Göttingen, West-Deutschland. Paläontologische Zeitschrift 60(1–2): 109–129.

Schwarz W (1970) *Birgeria stensioei* Aldinger. In: Kuhn-Schnyder E, Peyer B, editors. Die Triasfauna der Tessiner Kalkalpen, X. Schweizerische Paläontologische Abhandlungen, 89. pp. 1–93.

Stensiö E (1919) Einige Bemerkungen über die systematische Stellung von *Saurichthys mougeoti* Agassiz. Senckenbergiana 1(6): 177–181.

Stensiö E (1921) Triassic Fishes from Spitzbergen 1. Vienna: Adolf Holzhausen. 307 p.

Stensiö E (1925) Triassic fishes from Spitzbergen 2. Kungliga Svenska Vetenskapsakademiens Handlingar 3: 1–261.

Stensiö E (1932) Triassic fishes from East Greenland 1–2. Meddelelser om Grønland 83(3): 1–298.

Stolley E (1920) Beiträge zur Kenntnis der Ganoiden des deutschen Muschelkalks. Palaeontographica 63: 25–86.

Su D-Z, Li Z-C (1983) A new Triassic perleidid fish from Hubei, China. Vertebrata PalAsiatica 21(1): 9–16. [in Chinese with English summary]

Sun Z-Y, Tintori A, Lombardo C, Jiang D-Y, Hao W-C, et al. (2008) A new species of the genus *Colobodus* Agassiz, 1844 (Osteichthyes, Actinopterygii) from the Pelsonian (Anisian, Middle Triassic) of Guizhou, South China. Rivista Italiana di Paleontologia e Stratigrafia 114: 363–376.

Sun Z-Y, Tintori A, Jiang D-Y, Lombardo C, Rusconi M, et al. (2009) A new perleidiform (Actinopterygii, Osteichthyes) from the middle Anisian (Middle Triassic) of Yunnan, South China. Acta Geologica Sinica 83: 460–470.

Takai F (1976) On *Atherstonia madagascariensis*, a new species of palaeoniscoid fish from Madagascar. Proceedings of the Japan Academy 52: 25–28.

Tanner VM (1936) A study of Utah fossil fishes with the description of a new genus and species. Proceedings of the Utah Academy of Sciences, Arts and Letters 13: 81–89.

Tintori A (1998) *Ctenognathichthys bellottii* (de Alessandri, 1910): Nomenclatural problems and stratigraphical importance of this Middle Triassic actinopterygian fish. Rivista Italiana di Paleontologia e Stratigrafia 104(3): 417–423.

Tintori A, Lombardo C (1999) Late Ladinian fish faunas from Lombardy (North Italy): stratigraphy and paleobiology, In: Arratia G, Schultze H-P, eds) Mesozoic Fishes 2. Systematics and Fossil Record. München: Dr. Friedrich Pfeil. pp. 495–504.

Tintori A, Sun Z-Y, Lombardo C, Jiang D-Y, Sun Y-L, Rusconi M, Hao W-C (2008) New specialized basal neopterygians (Actinopterygii) from Triassic of the Tethys realm. Geologia Insubrica 2007(10/2): 13–20.

Tintori A, Sun Z-Y, Lombardo C, Jiang D-Y, Sun Y-L, Hao W-C (2010) A new basal neopterygian from the Middle Triassico f Luoping County (South China). Rivista Italiana di Paleontologia e Stratigrafia 116(2): 161–172.

Tong J, Zhou X, Erwin DH, Zuo J, Zhao L (2006) Fossil fishes from the Lower Triassic of Majiashan, Chaohu, Anhui Province, China. Journal of Paleontology 80: 146–161.

Wen W, Zhang Q-Y, Hu S-X, Zhou C-Y, Xie T, Huang J-Y, Chen ZQ, Benton MJ (2012) A new basal actinopterygian fish from the Anisian (Middle Triassic) of Luoping, Yunnan Province, Southwest China. Acta Palaeontologica Polonica 57(1): 149–160.

Wen W, Zhang Q-Y, Hu S-X, Benton MJ, Zhou C-Y, Tao X, Huang J-Y, Chen Z-Q (2013) Coelacanths from the Middle Triassic Luoping Biota, Yunnan, South China, with the earliest evidence of ovoviviparity. Acta Palaeontologica Polonica 58 (1): 175–193.

Wendruff AJ, Wilson MVH (2012) A fork-tailed coelacanth, *Rebellatrix divaricerca*, gen. et sp. nov. (Actinistia, Rebellatricidae, fam. nov.), from the Lower Triassic of Western Canada. Journal of Vertebrate Paleontology 32: 499–511.

White EI (1933) New Triassic palaeoniscids from Madagascar. Annals and Magazine of Natural History, Series 10, 11: 118–128.

White EI, Moy-Thomas MA (1940) XLVIII. — Notes on the nomenclature of fossil fishes, 2. Homonyms D–L, Journal of Natural History, Series 11, 6(31): 98–103.

White EI, Moy-Thomas MA (1941) XXV. — Notes on the nomenclature of fossil fishes, 3. Homonyms M–Z, Journal of Natural History, Series 11, 7(40): 395–400.

Wilser JL (1923) *Pygopterus crecelii* n. sp. aus dem Oberen Buntsandstein bei Karlsruhe i. B. Berichte der Naturforschenden Gesellschaft zu Freiburg i. Br., 23(2): 68–78.

Woodward AS (1895) Catalogue of the fossil fishes in the British Museum (Natural History), Part III. London: British Museum (Natural History). 544 p.

Woodward AS (1910) On some Permo-Carboniferous fishes from Madagascar. The Annals and Magazine of Natural History, Series 8, 5: 1–6.

Wu FX, Sun YL, Hao WC, Jiang DY, Xu GH, Sun ZY, Tintori A (2009) A new species of *Saurichthys* (Actinopterygii: Saurichthyidae) from Middle Triassic (Anisian) of Yunnan Province, China. Acta Geologica Sinica [English Edition] 83: 440–450.

Wu, FX, Sun, YL, Xu GH, Hao WC, Jiang DY, Sun ZY (2011) New saurichthyid actinopterygian fishes from the Anisian (Middle Triassic) of southwestern China. Acta Palaeontologica Polonica 56(3): 581–614.

Xu, G-H, Wu, F-X (2012) A deep-bodied ginglymodian fish from the Middle Triassic of eastern Yunnan Province, China, and the phylogeny of lower neopterygians. Chinese Science Bulletin 57(1): 111–118.

Zhang QY, Zhou CY, Lü T & Bai JK (2010) Discovery of Middle Triassic *Saurichthys* in the Luoping area, Yunnan, China. Geological Bulletin of China 29: 26–30. [in Chinese, with English abstract]

Zhao L-J, Lu L-W (2007) A new genus of Early Triassic perleidid fish from Changxing, Zhejiang, China. Acta Palaeontologica Sinica 46(2): 238–243. [in Chinese with English summary]

**References to Appendix S2:**

Agassiz L (1833-45) Recherches sur les Poissons Fossiles, Vol. I-V. Neuchâtel: Imprimaire de Petitpierre.

Albers PCH, Rieppel O (2003) A new species of the sauropterygian genus *Nothosaurus* from the Lower Muschelkalk of Winterswijk, The Netherlands. Journal of Paleontology 77: 738–744.

Arthaber GV (1924) Die Phylogenie der Nothosaurier. Acta Zoologica (Stockholm) 5: 439-516.

Bassani F (1886) Sui fossili e sull’età degli schisti bituminosi Triasici di Besano in Lombardia. Communicazione preliminare. Atti della Società Italiana di Scienze Naturale 29: 15–72.

Brinkmann W (1998a) Die Ichthyosaurier (Reptilia) aus der Grenzbitumenzone (Mitteltrias) des Monte San Giorgio (Tessin, Schweiz) - neue Ergebnisse. Vierteljahresschrift der Naturforschenden Gesellschaft in Zürich 143: 165–177.

Brinkmann W (1998b) *Sangiorgiosaurus* n. g. - eine neue Mixosaurier-Gattung (Mixosauridae, Ichthyosauria) mit Quetschzähnen aus der Grenzbitumenzone (Mitteltrias) des Monte San Giorgio (Schweiz, Kanton Tessin). Neues Jahrbuch für Geologie und Paläontologie, Abhandlungen 207: 125–144.

Brinkman DB, Zhao X, Nicholls EL (1992) A primitive ichthyosaur from the Lower Triassic of British Columbia, Canada. Palaeontology 35: 465–474.

Brotzen F (1956) Stratigraphical studies on the Triassic vertebrate fossils from Wadi Raman, Israel. Arkiv for Mineralogi och Geologi 2: 191-217.

Callaway JM, Brinkman D (1989) Ichthyosaurs (Reptilia, Ichthyosauria) from the Lower and Middle Triassic Sulphur Mountain Formation, Wapiti Lake area, British Columbia, Canada. Canadian Journal of Earth Sciences 26: 1491-1500.

Carroll RL, Dong Z-M (1991) *Hupehsuchus*, an enigmatic aquatic reptile from the Triassic of China, and the problem of establishing relationships. Philosophical Transactions of the Royal Society of London Series B 331: 131–153.

Carroll RL, Gaskill P (1985) The nothosaur *Pachypleurosaurus* and the origin of plesiosaurs. Philosophical Transactions of the Royal Society of London Series B 309: 343–393.

Case EC (1936) A nothosaur from the Triassic of Wyoming. University of Michigan Contributions from the Museum of Paleontology 5: 1–36.

Chen X, Sander PM, Cheng L, Wang X (2013) A new Triassic ichthyosaur from Yuanan, South China. Acta Geologica Sinica (English Edition) 87(3): 672–677.

Cheng Y-N, Wu X-C, Sato T, Shan H-Y (2012) A new eosauropterygian (Diapsida, Sauropterygia) from the Triassic of China. Journal of Vertebrate Paleontology 32: 1335–1349 [doi: 1310.1080/02724634.02722012.02695983].

Cornalia E ( 1854) Notizie zoologiche suI *Pachypleura edwardsii* Cor. Nuovo sauro acrodonte degli strati triasici di Lombardia. Giornale del' JR Instituto Lombardo di Scienze, Lettre, ed Arti, Nuova Serie 6: 1–46.

Cox CB, Smith DG (1973) A review of the Triassic vertebrate faunas of Svalbard. Geological Magazine 110: 405–418.

Curioni G ( 1847) Cenni sopra un nuovo saurio fossile dei monti di Perledo sul Lario e sul terreno che lo racchiude. Giornale dell' I R Istituto Lombardo di Scienze, Lettre ed Arti 16: 159–170.

Cuthbertson RS, Russell AP, Anderson JS (2013) Cranial morphology and relationships of a new grippidian (Ichthyopterygia) from the Vega-Phroso Siltstone Member (Lower Triassic) of British Columbia, Canada. Journal of Vertebrate Paleontology 33: 831–847 [doi: 810.1080/02724634.02722013.02755989].

Dalla Vecchia FM (2004) First record of the rare marine reptile *Tholodus schmidi* from the Middle Triassic of the Southern Alps. Rivista Italiana di Paleontologia e Stratigrafia 110: 479–492.

Dal Sasso C, Pinna G (1996 ) *Besanosaurus leptorhynchus* n. gen. n. sp., a new shastasaurid ichthyosaur from the MiddleTriassic of Besano (Lombardy, N. Italy). Paleontologia Lombarda Nuova serie 4: 3–23.

Dames W (1890) *Anarosaurus pumilio* nov. gen. nov. sp. Zeitschrift der deutschen Geologischen Gesellschaft 42: 74–85.

Drevermann F (1933) Die Placodontier. 3. Das Skelett von *Placodus gigas* Agassiz im Senckenberg-Museum. Abhandlungen der Senckenbergischen Naturforschenden Gesellschaft 38: 321–364.

Edinger T (1921) Über *Nothosaurus*. II. Zur Gaumenfrage. Senckenbergiana 3: 193–205.

Fraas O (1881) *Simosaurus pusillus* aus der Lettenkohle von Hoheneck. Jahreshefte des Vereins für vaterländische Naturkunde in Württemberg 37: 319–324.

Fraas E (1896) Die schwäbischen Trias-Saurier nach dem Material der Kgl. Naturalien-Sammlung in Stuttgart zusammengestellt. Festgabe des Königlichen Naturalien-Cabinets in Stuttgart zur 42. Versammlung der Deutschen geologischen Gesellschaft in Stuttgart, August 1896. Stuttgart: E. Schweizerbart'sche Verlagshandlung (E. Koch). 18 p.

Fritsch KV (1894) Beitrag zur Kenntnis der Saurier des Halle'schen unteren Muschelkalkes. Abhandlungen der Naturforschenden Gesellschaft zu Halle 20: 273–302.

Fröbisch NB, Sander PM, Rieppel O (2006) A new species of *Cymbospondylus* (Diapsida, Ichthyosauria) from the Middle Triassic of Nevada and a re-evaluation of the skull osteology of the genus. Zoological Journal of the Linnean Society 147: 515–538.

Geissler G (1895) Ueber neue Saurier-Funde aus dem Muschelkalk von Bayreuth. Zeitschrift der Deutschen Geologischen Gesellschaft 47: 331–355.

Gürich GJE (1884) Über einige Saurier des oberschlesischen Muschelkalkes. Zeitschrift der Deutschen Geologischen Gesellschaft 36: 125–144.

Haas G (1959) On some fragments of the dermal skeleton of Placodontia from the Trias of Aarif en Naqa, Sinai Peninsula. Kunglia Svenska vetenskapsakademiens Handlingar Fjärde Serien 7: 1–19.

Haas G (1963) *Micronothosaurus stensiöi*, ein neuer Nothosauride aus dem Oberen Muschelkalk des Wadi Ramon, Israel. Palaontologische Zeitschrift 37: 161–178.

Haas G (1980) Ein Nothosaurier-Schädel aus dem Muschelkalk des Wadi Ramon (Negev, Israel). Annalen des Naturhistorischen Museums in Wien 83: 119-125.

Hänni K (2004) Die Gattung *Ceresiosaurus*. *Ceresiosaurus* *calgagnii* Peyer und *Ceresiosaurus* *lanzi* n.sp. (Lariosauridae, Sauropterygia). Zürich: University of Zürich. 146 p.

Huene Fv (1905) Über die Trias-Dinosaurier Europas. Zeitschrift der Deutschen Geologischen Gesellschaft 57: 345–349.

Huene Fv (1920) *Gonioglyptus*, ein alttriassischer Stegocephale aus Indien. Acta Zoologica (Stockholm) 1: 433–464.

Huene Fv (1958) Aus den Lechtaler Alpen ein neuer *Anarosaurus*. Neues Jahrbuch für Geologie und Paläontologie, Monatshefte 8/9: 382–384.

Jiang D-y, Maisch MW, Sun Z-y, Sun Y-l, Hao W-c (2006a) A new species of *Lariosaurus* (Reptilia, Sauropterygia) from the Middle Anisian (Middle Triassic) of southwestern China. Neues Jahrbuch fuer Geologie und Paläontologie, Abhandlungen 242: 19–42.

Jiang D-y, Maisch MW, Hao W-c, Sun Y-l, Sun Z-y (2006b) *Nothosaurus yangjuanensis* n. sp. (Reptilia, Sauropterygia, Nothosauridae) from the middle Anisian (Middle Triassic) of Guizhou, southwestern China. Neues Jahrbuch für Geologie und Paläontologie, Monatshefte 5: 257–276.

Jiang D-Y, Schmitz L, Hao W-C, Sun Y-L (2006c) A new mixosaurid ichthyosaur from the Middle Triassic of China. Journal of Vertebrate Paleontology 26: 60–69.

Jiang D-Y, Motani R, Hao W-C, Rieppel O, Sun Y-L, Schmitz L, Sun Z-Y (2008a) First record of Placodontoidea (Reptilia, Sauropterygia, Placodontia) from the Eastern Tethys. Journal of Vertebrate Paleontology 28: 904–908.

Jiang D-Y, Rieppel O, Motani R, Hao W-C, Sun Y-L, et al. (2008b) A new Middle Triassic eosauropterygian (Reptilia, Sauropterygia) from Southwestern China. Journal of Vertebrate Paleontology 28: 1055-1062 [doi: 1010.1671/0272-4634-1028.1054.1055].

Jiang D, Motani R, Hao W, Schmitz L, Rieppel O, et al. (2008c) New primitive ichthyosaurian (Reptilia, Diapsida) from the Middle Triassic of Panxian, Guizhou, southwestern China and its position in the Triassic biotic recovery. Progress in Natural Science 18: 1315-1319 [doi:1310.1016/j.pnsc.2008.1301.1039].

Jiang D-Y, Motani R, Tintori A, Rieppel O, Sun Z-Y (2012) Two new Early Triassic marine reptiles from Chaohu, Anhui Province, South China. Journal of Vertebrate Paleontology, SVP Program and Abstracts Book, 2012: 117.

Kelley NP, Motani R, Jiang D-y, Rieppel O, Schmitz A (2012) Selective extinction of Triassic marine reptiles during long-term sea-level changes illuminated by seawater strontium isotopes. Palaeogeography, Palaeoclimatology, Palaeoecology [doi: 10.1016/j.palaeo.2012.1007.1026].

Klein N (2009) Skull morphology of Anarosaurus heterodontus (Reptilia: Sauropterygia: Pachypleurosauria) from the lower Muschelkalk of the Germanic Basin (Winterswijk, the Netherlands) Journal of Vertebrate Paleontology 29: 665–676 [doi: 610.1671/1039.1029.0327].

Klein N (2012) Postcranial morphology and growth of the pachypleurosaur *Anarosaurus heterodontus* (Sauropterygia) from the Lower Muschelkalk of Winterswijk, The Netherlands. Paläontologische Zeitschrift 86: 389–408 [doi: 310.1007/s12542-12012-10137-12541].

Klein N, Albers PCH (2009) A new species of the sauropsid reptile *Nothosaurus* from the Lower Muschelkalk of the western Germanic Basin, Winterswijk, The Netherlands. Acta Palaeontologica Polonica 54: 589–598 [doi: 510.4202/app.2008.0083].

Klein N, Scheyer TM (2013) A new placodont sauropterygian from the Middle Triassic of the Netherlands. Acta Palaeolontologica Polonica [doi: 10.4202/app.2012.0147].

Koken E (1893) Beiträge zur Kenntnis der Gattung *Nothosaurus*. Zeitschrift der Deutschen Geologischen Gesellschaft 45: 337–377.

Kuhn E (1942) Über einen weiteren Fund von *Paraplacodus broilii* Peyer aus der Trias des Monte San Giorgio. Eclogae geologicae Helvetiae 35: 174–183.

Kuhn-Schnyder E (1959) Ein neuer Pachypleurosaurier von der Stulseralp bei Bergün (Kt. Graubünden, Schweiz). Eclogae geologicae Helvetiae 52: 639–658 [doi: 610.5169/seals-162592].

Kuhn O (1961) Die Familien der rezenten und fossilen Amphibien und Reptilien. Verlagshaus Meisenbach KG, Bamberg. 79p.

Kuhn-Schnyder E (1987) Die Triasfauna der Tessiner Kalkalpen. XXVI. *Lariosaurus lavizzarii* n. sp. (Reptilia, Sauropterygia). Schweizerische Paläontologische Abhandlungen 110: 1–24.

Lehman J-P (1961) Les Stégocéphales de Madagascar. Annales de Paléontologie 47: 42–46.

Leidy J (1868) Notice of some reptilian remains from Nevada. Proceedings of the Philadelphia Academy of Sciences 20: 177–178.

Li C (2003) First record of protorosaurid reptile (Order Protorosauria) from the Middle Triassic of China. Acta Geologica Sinica 77: 419-423.

Li J-L, Rieppel O (2004) A new nothosaur from Middle Triassic of Guizhou, China. Vertebrata PalAsiatica 42: 1-12.

Li J-L, Liu J, Li C, Huang Z-X (2002) The horizon and age of the marine reptiles from Hubei Province, China [in Chinese with English translation]. Vertebrata PalAsiatica 40: 241–244.

Li C, Jiang D-Y, Cheng L, Wu X-C, Rieppel O (2013) A new species of Largocephalosaurus (Diapsida: Saurosphargidae), with implications for the morphological diversity and phylogeny of the group. Geological Magazine [doi: 10.1017/S001675681300023X].

Li C, Rieppel O, LaBarbera MC (2004) A Triassic aquatic protorosaur with an extremely long neck. Science 305: 1931.

Li C, Rieppel O, Wu X-C, Zhao L-J, Wang L-T (2011) A new Triassic marine reptile from southwestern China. Journal of Vertebrate Paleontology 31: 303–312 [doi: 310.1080/02724634.02722011.02550368].

Li C, Wu X-c, Cheng Y-n, Sato T, Wang L (2006) An unusual archosaurian from the marine Triassic of China. Naturwissenschaften 93: 200–206.

Liu J, Rieppel O, Jiang D-Y, Aitchison JC, Motani R, et al. (2011) A new pachypleurosaur (Reptilia: Sauropterygia) from the lower Middle Triassic of southwestern China and the phylogenetic relationships of Chinese pachypleurosaurs. Journal of Vertebrate Paleontology 31: 292–302 [doi: 210.1080/02724634.02722011.02550363].

Liu J, Motani R, Jiang D-Y, Hu S-X, Aitchison JC, et al. (2013) The first specimen of the Middle Triassic *Phalarodon atavus* (Ichthyosauria: Mixosauridae) from South China, showing postcranial anatomy and peri-Tethyan distribution. Palaeontology 56: 849–866 [doi: 810.1111/pala.12021].

Maganuco S, Steyer JS, Pasini G, Boulay M, Lorrain S, et al. (2009) An exquisite specimen of *Edingerella madagascariensis* (Temnospondyli) from the Lower Triassic of NW Madagascar; cranial anatomy, phylogeny, and restorations. Memorie della Società Italiana di Scienze Naturali e del Museo Civico di Storia Naturale di Milano 36: 1–72.

Maisch MW (2010) Phylogeny, systematics, and origin of the Ichthyosauria – the state of the art. Palaeodiversity 3: 151–214.

Maisch MW, Matzke AT (2000) The Ichthyosauria. Stuttgarter Beiträge zur Naturkunde, Serie B (Geologie und Paläontologie) 298: 1–159.

Maisch MW, Matzke AT (2003) Observations on Triassic ichthyosaurs. Part XII. A new Early Triassic ichthyosaur genus from Spitzbergen. Neues Jahrbuch für Geologie und Paläontologie, Abhandlungen 229: 317–338.

Mariani E (1923) Su un nuovo esemplare di *Lariosaurus balsami* Cur. trovato negli scisti di Perledo sopra Varenna (Lago di Como). Atti della Societa Italiana di Scienze Naturali 62: 218–225.

Massare JA, Callaway JM (1994) *Cymbospondylus* (Ichthyosauria: Shastasauridae) from the Lower Triassic Thaynes Formation of Southeastern Idaho. Journal of Vertebrate Paleontology 14: 139–141.

Maxwell EE, Kear BP (2013) Triassic ichthyopterygian assemblages of the Svalbard Archipelago: a reassessment of taxonomy and distribution. GFF 135: 85–94 [doi: 10.1080/11035897.11032012.11759145].

Mazin J-M (1981) *Svalbardosaurus crassidens* n.g., n.sp., un Ichthyopterygien nouveau du Spathian (Trias inferieur) du Spitzberg. Comptes Rendus de l’Académie des Sciences, Paris 293 (2): 203–205.

Mazin J-M, Bucher H (1987) *Omphalosaurus nettarhynchus*, une nouvelle espèce d'Omphalosauridé (Reptilia, Ichthyopterygia) du Spathien de la Humboldt Range (Nevada, U.S.A.). Comptes Rendus de l'Academie des Sciences, Paris, Série II 305: 823–828.

Mazin J-M, Suteethorn V, Buffetaut E, Jaeger J-J, Helmcke-Ingavat R (1991) Preliminary description of *Thaisaurus chonglakmanii* n. g., n. sp., a new ichthyopterygian (Reptilia) from the Early Triassic of Thailand. Comptes Rendus de l'Academie des Sciences Paris Série II 313: 1207–1212.

McGowan C, Motani R (2003) Ichthyopterygia. Handbuch der Paläoherpetologie [Handbook of Paleoherpetology]. München, Germany: F. Pfeil. Part 8: 173p.

Merriam JC (1906) Preliminary note on a new marine reptile from the Middle Triassic of Nevada. Bulletin of the Department of Geology at the University of California 5: 75–79.

Merriam JC (1910) The skull and dentition of a primitive ichthyosaurian from the Middle Triassic. University of California Publications, Bulletin of the Department of Geology at the University of California 5: 381–390.

Meyer Hv (1842) *Simosaurus*, die Stumpfschnauze, ein Saurier aus dem Muschelkalke von Luneville. Neues Jahrbuch für Mineralogie, Geognosie, Geologie und Petrefaktenkunde 1842: 184–197.

Meyer Hv (1947-1955) Die Saurier des Muschelkalks mit Rücksicht auf die Saurier aus Buntem Sandstein und Keuper. Zur Fauna der Vorwelt, zweite Abtheilung, VIII+ 167 pages. Frankfurt: Heinrich Keller.

Motani R (1997) Phylogeny of the Ichthyosauria (Amniota: Reptilia) with special reference to Triassic forms. PhD Thesis, Graduate Department of Zoology, Toronto: University of Toronto. 384 p.

Motani R (1998) First complete forefin of the ichthyosaur *Grippia longirostris* from the Triassic of Spitzbergen. Palaeontology 41 (4): 591–599.

Motani R (2000) Is *Omphalosaurus* ichthyopterygian?--A phylogenetic perspective. Journal of Vertebrate Paleontology 20: 295–301 [doi: 210.1671/0272-4634(2000)1020[0295:IOIAPP]1672.1670.CO;1672].

Motani R, You H (1998) Taxonomy and limb ontogeny of *Chaohusaurus geishanensis* (Ichthyosauria), with a note on the allometric equation. Journal of Vertebrate Paleontology 18: 533–540.

Motani R, Minoura N, Ando T (1998) Ichthyosaurian relationships illuminated by new primitive skeletons from Japan. Nature 393: 255–257.

Motani R, You H, McGowan C (1996) Eel-like swimming in the earliest ichthyosaurs. Nature 382: 347–348 [doi:310.1038/382347a382340].

Müller J (2005) The anatomy of *Askeptosaurus italicus* from the Middle Triassic of Monte San Giorgio and the interrelationships of thalattosaurs (Reptilia, Diapsida). Canadian Journal of Earth Sciences 42: 1347–1367 [doi:1310.1139/e1305-1030].

Münster G (1834) Vorläufige Nachricht über einige neue Reptilien im Muschelkalke von Baiern. Neues Jahrbuch für Mineralogie, Geognosie, Geologie und Petrefaktenkunde 1834: 521-527.

Münster G (1839) Beiträge zur Petrefaktenkunde, mit XVIII nach der Natur gezeichneten Tafeln der Herren Hermann v. Meyer und Professor Rudolph Wagner. Bayreuth: Buchner'sche Buchhandlung.

Neenan JM, Klein N, Scheyer TM (2013) European origin of placodont marine reptiles and the evolution of crushing dentition in Placodontia. Nature Communications 4:1621 [doi: 10.1038/ncomms2633].

Nicholls EL (1999) A reexamination of *Thalattosaurus* and *Nectosaurus* and the relationships of the Thalattosauria (Reptilia: Diapsida). PaleoBios 19: 1–29.

Nicholls EL, Brinkman DB (1993a) New thalattosaurs (Reptilia: Diapsida) from the Triassic Sulphur Mountain Formation of Wapiti Lake, British Columbia. Journal of Paleontology 67: 263–278.

Nicholls EL, Brinkman D (1993B) A new specimen of *Utatsusaurus* (Reptilia: Ichthyosauria) from the Lower Triassic Sulphur Mountain Formation of British Columbia. Canadian Journal of Earth Sciences 30: 486–490.

Nicholls EL, Brinkman DB (1995) A new ichthyosaur from the Triassic Sulphur Mountain Formation of British Columbia. In: Sarjeant WAS, editor. Vertebrate Fossils and the Evolution of Scientific Concepts. Amsterdam: Gordon and Breach Publishers. pp. 521–535.

Nicholls EL, Brinkman DB, Callaway JM (1999) New material of *Phalarodon* (Reptilia: Ichthyosauria) from the Triassic of British Columbia and its bearings on the interrelationships of mixosaurs. Palaeontographica, Abteilung A 252: 1–22.

Nilsson T (1942) Über einige postkraniale Skelettreste des triassichen Stegocephalen Spitsbergens. Bulletin of the Geological Institution of the University of Uppsala 30: 227–272.

Nopcsa FB (1925) *Askeptosaurus*, ein neues Reptil der Trias von Besano. Centralblatt für Mineralogie, Geologie und Paläontologie, Abt B: Geologie und Paläontologie 1925: 265–268.

Nopcsa F (1929) On some nothosaurian reptiles from the Trias. Palaeontological Notes on Reptiles. Geologica Hungarica - Series Palaeontologica Tomus 1 (Fasc 1) [84pp]. pp. 20–44.

Nosotti S (2007) *Tanystropheus* *longobardicus* (Reptilia, Protorosauria): re-interpretations of the anatomy based on new specimens from the Middle Triassic of Besano (Lombardy, Northern Italy). Memoire della Società Italiana di Scienze Naturali e del Museo Civico di Storia Naturale di Milano 35: 1–88.

Nosotti S, Pinna G (1993) *Cyamodus kuhn-schnyderi* n. sp., nouvelle espèce de Cyamodontidae (Reptilia, Placodontia) du Muschelkalk supérieur allemand. Comptes Rendus de l'Academie des Sciences Paris Série II 317: 847–850.

Nosotti S, Rieppel O (2003) *Eusaurosphargis dalsassoi* n. gen n. sp., a new, unusual diapsid reptile from the Middle Triassic of Besano (Lombardy, N Italy). Memorie della Società Italiana di Scienze Naturali e del Museo Civico di Storia Naturale di Milano 31: 3–33.

Peyer B (1931a) Die Triasfauna der Tessiner Kalkalpen. IV. *Ceresiosaurus calcagnii* nov. gen. nov. spec. Abhandlungen der Schweizerischen Paläontologischen Gesellschaft 51: 1–68.

Peyer B (1931b) Die Triasfauna der Tessiner Kalkalpen III. Placodontia. Abhandlungen der schweizerischen Paläontologischen Gesellschaft 51: 1–25.

Peyer B (1931c) *Paraplacodus broilii* nov. gen. nov. sp., ein neuer Placodontier aus der Tessiner Trias. Centralblatt für Mineralogie, etc Abt B 10: 570–573.

Peyer B (1936a) Die Triasfauna der Tessiner Kalkalpen. X. *Clarazia schinzi* nov. gen. nov. spec. Abhandlungen der schweizerischen Paläontologischen Gesellschaft 57: 1–61.

Peyer B (1936b) Die Triasfauna der Tessiner Kalkalpen. XI. Hescheleria rübeli nov. gen. nov. spec. Abhandlungen der schweizerischen Paläontologischen Gesellschaft 58: 1–48.

Peyer B (1939) Die Triasfauna der Tessiner Kalkalpen XIV. *Paranothosaurus amsleri* nov. gen. nov. spec. Abhandlungen der Schweizerischen Paläontologischen Gesellschaft 62: 1–87.

Peyer B (1955) Die Triasfauna der Tessiner Kalkalpen XVIII. *Helveticosaurus* *zollingeri* n. g. n. sp. Abhandlungen der schweizerischen Paläontologischen Gesellschaft 72: 4–50.

Quenstedt FA (1852) Handbuch der Petrefaktenkunde. Tübingen: H. Laupp. 792 p.

Renesto S, Pareo M, Lombardo C (2004) A new specimen of the sauropterygian reptile *Lariosaurus* from the Kalkschieferzone (Uppermost Ladinian) of Valceresio (Varese N. Italy). Neues Jahrbuch für Geologie und Paläontologie, Monatshefte 2004(6): 351–369.

Rieppel O (1989a) A new pachypleurosaur (Reptilia: Sauropterygia) from the Middle Triassic of Monte San Giorgio, Switzerland. Philosophical Transactions of the Royal Society of London Series B - Biological Sciences 323: 1–73.

Rieppel O (1989b) *Helveticosaurus zollingeri* Peyer (Reptilia, Diapsida) skeletal paedomorphosis, functional anatomy and systematic affinities. Palaeontographica, Abt A 208: 123–152.

Rieppel O (1994) Osteology of *Simosaurus gaillardoti* and the relationships of stem-group Sauropterygia. Fieldiana (Geology), New Series 28: 1–85.

Rieppel O (1997) Revision of the sauropterygian reptile genus *Cymatosaurus* v. Fritsch, 1894, and the relationships of *Germanosaurus* Nopcsa, 1928, from the Middle Triassic of Europe. Fieldiana: Geology, New Series 36: 1–38.

Rieppel O (1998a) *Corosaurus alcovensis* Case and the phylogenetic interrelationships of Triassic stem-group Sauropterygia. Zoological Journal of the Linnean Society 124: 1–41.

Rieppel O (1998b) The systematic status of *Hanosaurus hupehensis* (Reptilia, Sauropterygia) from the Triassic of China. Journal of Vertebrate Paleontology 18 (3): 545–557 [doi: 10.1080/02724634.1998.10011082].

Rieppel O (1998c) The status of the sauropterygian reptile genera *Ceresiosaurus*, *Lariosaurus*, and *Silvestrosaurus* from the Middle Triassic of Europe. Fieldiana (Geology), New Series 38: 1–46.

Rieppel O (1999) The sauropterygian genera *Chinchenia*, *Kwangsisaurus*, and *Sanchiaosaurus* from the Lower and Middle Triassic of China. Journal of Vertebrate Paleontology 19: 321–337 [doi: 310.1080/02724634.02721999.10011144].

Rieppel O (2000) Sauropterygia I - Placodontia, Pachypleurosauria, Nothosauroidea, Pistosauroidea. Handbuch der Paläoherpetologie [Handbook of Paleoherpetology]. München, Germany: F. Pfeil. Part 12A: 134p.

Rieppel O (2001a) A new species of *Nothosaurus* (Reptilia: Sauropterygia) from the Upper Muschelkalk (Lower Ladinian) of southwestern Germany. Palaeontographica, Abt A 263: 137–161.

Rieppel O (2001b) A new species of *Tanystropheus* (Reptilia: Protorosauria) from the Middle Triassic of Makhtesh Ramon, Israel. Neues Jahrbuch für Geologie und Paläontologie, Abhandlungen 221: 271–287.

Rieppel O (2002) The dermal armor of the cyamodontoid placodonts (Reptilia, Sauropterygia): morphology and systematic value. Fieldiana: Geology, New Series 46: 1–41.

Rieppel O, Dalla Vecchia FM (2001) Marine reptiles from the Triassic of the Tre Venezie Area, Northeastern Italy. Fieldiana: Geology, New Series 44: 1–25.

Rieppel O, Hagdorn H (1997) Chapter 5. Paleobiogeography of Middle Triassic Sauropterygia in central and western Europe. In: Callaway JM, Nicholls EL, editors. Ancient Marine Reptiles. San Diego, California: Academic Press. pp. 121–144.

Rieppel O, Hagdorn H (1998) Fossil reptiles from the Spanish Muschelkalk (Mont-Ral and Alcover, Province Tarragona). Historical Biology 13: 77–97 [doi: 10.1080/08912969809386575].

Rieppel O, Lin K (1995) Pachypleurosaurs (Reptilia: Sauropterygia) from the Lower Muschelkalk, and a review of the Pachypleurosauroidea. Fieldiana (Geology), New Series 32: 1–44.

Rieppel O, Werneburg R (1998) A new species of the sauropterygian Cymatosaurus from the Lower Muschelkalk of Thuringia, Germany. Palaeontology 41: 575–589.

Rieppel O, Wild R (1996) A revision of the genus *Nothosaurus* (Reptilia: Sauropterygia) from the Germanic Triassic, with comments on the status of Conchiosaurus clavatus. Fieldiana (Geology), New Series No. 34: 1–82.

Rieppel O, Li C, Fraser NC (2008) The skeletal anatomy of the Triassic protorosaur Dinocephalosaurus orientalis Li, from the Middle Triassic of Guizhou Province, Southern China. Journal of Vertebrate Paleontology 28: 95–110.

Rieppel O, Liu J, Li C (2006) A new species of the thalattosaur genus *Anshunsaurus* (Reptilia: Thalattosauria) from the Middle Triassic of Guizhou Province, Southwestern China. Vertebrata PalAsiatica 44: 285–296.

Rieppel O, Mazin J-M, Tchernov E (1997) Speciation along rifting continental margins: a new nothosaur from the Negev (Israël). Comptes Rendus de l'Academie des Sciences Paris, Sciences de la Terre et des Planètes / Earth and Planetary Sciences [Série II a] 325: 991–997.

Rieppel O, Mazin J-M, Tchernov E (1999) Sauropterygia from the Middle Triassic of Makhtesh Ramon, Negev, Israel. Fieldiana (Geology), New Series 40: 1–85.

Rieppel O, Jiang D-Y, Fraser NC, Hao W-C, Motani R, et al. (2010) *Tanystropheus* cf. *T. longobardicus* from the early Late Triassic of Guizhou Province, southwestern China Journal of Vertebrate Paleontology 30: 1082–1089 [doi: 1010.1080/02724634.02722010.02483548].

Sander PM (1989a) The pachypleurosaurids (Reptilia: Nothosauria) from the Middle Triassic of Monte San Giorgio (Switzerland) with the description of a new species. Philosophical Transactions of the Royal Society of London Series B - Biological Sciences 325: 561–666 [doi: 510.1098/rstb.1989.0103].

Sander PM (1989b) The large ichthyosaur *Cymbospondylus buchseri*, sp. nov., from the Middle Triassic of Monte San Giorgio (Switzerland), with a survey of the genus in Europe. Journal of Vertebrate Paleontology 9: 163–173.

Sander PM (1997) The paleobiogeography of *Shastasaurus*. In: Callaway JM, Nicholls EL, editors. Ancient Marine Reptiles. London: Academic Press. pp. 317–343.

Sander PM, Faber C (2003) The Triassic marine reptile *Omphalosaurus*: osteology, jaw anatomy, and evidence for ichthyosaurian affinities. Journal of Vertebrate Paleontology 23: 799–816 [doi: 710.1671/1676].

Sander PM, Rieppel OC, Bucher H (1997) A new pistosaurid (Reptilia: Sauropterygia) from the Middle Triassic of Nevada and its implications for the origin of the plesiosaurs. Journal of Vertebrate Paleontology 17: 526–533.

Sanz JL (1983 ) Los Nothosaurios (Reptilia, Sauropterygia) Espanoles. Estudios geológicos 39: 193–215.

Sato T, Cheng Y-N, Wu X-C, Shan H-Y (2013a) *Diandongosaurus acutidentatus* Shang, Wu & Li, 2011 (Diapsida: Sauropterygia) and the relationships of Chinese eosauropterygians. Geological Magazine [doi: 10.1017/S0016756813000356].

Sato T, Zaho L-J, Wu X-C, Li C (2013) A new specimen of the Triassic pistosauroid *Yunguisaurus*, with implications for the origin of Plesiosauria (Reptilia, Sauropterygia). Palaeontology [doi: 10.1111/pala.12048].

Säve-Söderbergh G (1935) On the dermal bones of the head in labyrinthodont stegocephalians and primitive Reptilia. Meddelelser om Grønland 98: 1–211.

Scheyer TM (2010) New interpretation of the postcranial skeleton and overall body shape of the placodont *Cyamodus hildegardis* Peyer, 1931 (Reptilia, Sauropterygia). Palaeontologia Electronica Vol. 13, Issue 2; 15A:15p; http://palaeo-electronica.org/2010_2/232/index.html.

Schmitz L (2005) The taxonomic status of *Mixosaurus nordenskioeldii*. Journal of Vertebrate Paleontology 25: 983–985.

Schmitz L, Sander PM, Storrs GW, Rieppel O (2004) New Mixosauridae (Ichthyosauria) from the Middle Triassic of the Augusta Mountains (Nevada, USA) and their implications for mixosaur taxonomy. Palaeontographica Abt A 270: 133–162.

Schoch RR, Milner AR (2000) Stereospondyli. Handbuch der Paläoherpetologie [Handbook of Paleoherpetology]. München, Germany: F. Pfeil. Part 3B: 220p.

Schröder H (1914) Wirbeltiere der Rüdersdorfer Trias. Abhandlungen der Königlich Preussischen Geologischen Landesanstalt, Neue Folge 65: 1–98.

Schultze H-P (1970) Über *Nothosaurus*. Neubeschreibung eines Schädels aus dem Keuper. Senckenbergiana lethaea 51: 211–237.

Seeley H (1882) On *Neusticosaurus pusillus* (Fraas), an amphibious reptile having affinities with terrestrial Nothosauria and with marine Plesiosauria. Quarterly Journal of the geological Society of London 38: 350–366.

Sennikov AG (2011) New tanystropheids (Reptilia: Archosauromorpha) from the Triassic of Europe. Paleontological Journal 45: 90–104 [Original Russian Text © A.G. Sennikov, 2011, published in Paleontologicheskii Zhurnal, 2011, No. 2011, pp. 2082–2096].

Shang Q-H (2006) A new species of *Nothosaurus* from the early Middle Triassic of Guizhou, China. Vertebrata PalAsiatica 44: 237–244.

Shang Q-H, Wu X-C, Li C (2011) A new eosauropterygian from the Middle Triassic of eastern Yunnan Province, southwestern China. Vertebrata PalAsiatica 49: 155–171.

Shikama T, Kamei T, Murata M (1978) Early Triassic ichthyosaurus, *Utatsusaurus hataii* gen. et sp. nov., from the Kitakami Massif, Northeast Japan. Tohoku University, Science Report, 2nd Series (Geology) 48: 77–97.

Shishkin MA (1980) The Luzocephalidae, a new Triassic labyrinthodont family [in Russian]. Paleontologicheskiy Zhurnal [Paleontological Journal] 14: 104–119.

Steyer JS (2002) The first articulated trematosaur 'amphibian' from the Lower Triassic of Madagascar: implications for the phylogeny of the group. Palaeontology 45: 771–793.

Steyer JS, Boulay M, Lorrain S (2010) 3D external restorations of stegocephalian skulls using ZBrush: the renaissance of fossil amphibians. Comptes Rendus Palevol 9: 463–470 [doi: 410.1016/j.crpv.2010.1007.1007].

Sues H-D, Carroll RL (1985) The pachypleurosaurid *Dactylosaurus schroederi* (Diapsida: Sauropterygia. Canadian Journal of Earth Sciences 22: 1602–1608.

Tichy G (1995) Ein früher, durophager Ichthyosaurier (Omphalosauridae) aus der Mitteltrias der Alpen. Geologisch-Paläontologische Mitteilungen, Innsbruck 20: 349–369.

Tintori A, Renesto S (1990) A new *Lariosaurus* from the Kalkschieferzone (uppermost Ladinian) of Valceresio (Varese, N. Italy). Bollettino della Società Paleontologica Italiana 29: 309–319.

Tschanz K (1989) *Lariosaurus buzzii* n. sp. from the Middle Triassic of Monte San Giorgio (Switzerland) with comments on the classification of nothosaurs. Palaeontographica Abt A 208: 153–179.

Wang K (1959) Ueber eine neue fossile Reptilform von Provinz Hupeh, China. Acta Palaeontologica Sinica 7: 373–378.

Watson DMS (1958) A new labyrinthodont (*Paracyclotosaurus*) from the upper Trias of New South Wales. Bulletin of the British Museum (Natural History), Geology 3: 233–263.

Welles SP (1993) A review of the lonchorhynchine trematosaurs (Labyrinthodontia), and a description of a new genus and species from the lower Moenkopi Formation of Arizona. PaleoBios 14: 1–24.

Wiman C (1910) Ichthyosaurier aus der Trias Spitzbergens. Bulletin of the Geological Institution of the University of Upsala 10 (1910-1911); 124–148.

Wiman C (1913) Über das Hinterhaupt der Labyrinthodonten. Bulletin of the Geological Institution of the University of Upsala 12: 1–8.

Wiman C (1914) Über die Stegocephalen aus der Trias Spitzbergens. Bulletin of the Geological Institution of the University of Upsala 13 (1915-1916): 1–34.

Wiman C (1916) Neue Stegocephalenfunde aus dem Posidonomyaschiefer Spitzbergens. Bulletin of the Geological Institution of the University of Upsala 13: 209–222.

Wiman C (1929) Eine neue Reptilien-Ordnung aus der Trias Spitzbergens. Bulletin of the Geological Institutions of the University of Upsala 22: 183–196.

Wild R (1974) E. Kuhn-Schnyder und B. Peyer: Die Triasfauna der Tessiner Kalkalpen. XXIII. *Tanystropheus longobardicus* (Bassani) (Neue Ergebnisse). Schweizerische Paläontologische Abhandlungen 95: 1–162.

Woodward AS (1904) On two new labyrinthodont skulls of the genera *Capitosaurus* and *Aphaneramma*. Proceedings of the Zoological Society, London No. 2: 170–176.

Wu X-C, Li Z, Zhou B-C, Dong Z-M (2003) A polydactylous amniote from the Triassic period. Nature 426: 516.

Wu X-C, Cheng Y-N, Li C, Zhao L-J, Sato T (2011) New information on *Wumengosaurus delicatomandibularis* Jiang et al., 2008 (Diapsida: Sauropterygia), with a revision of the osteology and phylogeny of the taxon. Journal of Vertebrate Paleontology 31: 70–83 [doi: 10.1080/02724634.02722011.02546724].

Young CC (1958) On the new Pachypleurosauroidea from Keichow, South-West China. Vertebrata PalAsiatica 2: 69–81.

Young CC (1959) On a new Nothosauria from the Lower Triassic beds of Kwangsi. Vertebrata PalAsiatica 3 (2): 73–79.

Young CC (1965) On the new nothosaurs from Hupeh and Kweichou, China. Vertebrata PalAsiatica 9: 337–356.

Young CC (1972) A marine lizard from Nanchang, Hupeh province. Memoirs of the Institute of Vertebrate Paleontology and Paleoanthropology, Academia Sinica 9: 17–28.

Young CC, Dong ZM (1972) On the Triassic aquatic reptiles of China. Institute of Vertebrate Paleontology and Palaeoanthropology, Chinese academy of Science, memoir 9: 1–34.

Zhao L-J, Li C, Liu J, He T (2008) A new armored placodont from the Middle Triassic of Yunnan Province, southwestern China. Vertebrata PalAsiatica 46: 171–177.
